# A meta-analysis on age-associated changes in blood DNA methylation: results from an original analysis pipeline for Infinium 450k data

**DOI:** 10.18632/aging.100718

**Published:** 2015-01-11

**Authors:** Maria Giulia Bacalini, Alessio Boattini, Davide Gentilini, Enrico Giampieri, Chiara Pirazzini, Cristina Giuliani, Elisa Fontanesi, Daniel Remondini, Miriam Capri, Alberto Del Rio, Donata Luiselli, Giovanni Vitale, Daniela Mari, Gastone Castellani, Anna Maria Di Blasio, Stefano Salvioli, Claudio Franceschi, Paolo Garagnani

**Affiliations:** ^1^ Department of Experimental, Diagnostic and Specialty Medicine, University of Bologna, Bologna 40138, Italy; ^2^ Interdepartmental Center “L. Galvani”, University of Bologna, Bologna 40126, Italy; ^3^ Personal Genomics S.r.l., Verona 37134, Italy; ^4^ Department of Biological, Geological and Environmental Sciences, University of Bologna, Bologna 40126, Italy; ^5^ Centro di Ricerche e Tecnologie Biomediche, Istituto Auxologico Italiano IRCCS, Cusano Milanino 20095, Italy; ^6^ Department of Physics and Astronomy, University of Bologna, Bologna 40126, Italy; ^7^ Institute of Organic Synthesis and Photoreactivity (ISOF) National Research Council (CNR), Bologna 40126, Italy; ^8^ Department of Clinical Sciences and Community Health, University of Milan, Milan, Italy; ^9^ Geriatric Unit, IRCCS Ca’ Granda Foundation Maggiore Policlinico Hospital, Milan, Italy; ^10^ IRCCS Institute of Neurological Sciences, Bologna, Italy; ^11^ Applied Biomedical Research Center, S. Orsola-Malpighi Polyclinic, Bologna 40138, Italy

**Keywords:** DNA methylation, Infinium HumanMethylation450 BeadChip, Epigenetics, aging

## Abstract

Aging is characterized by a profound remodeling of the epigenetic architecture in terms of DNA methylation patterns. To date the most effective tool to study genome wide DNA methylation changes is Infinium HumanMethylation450 BeadChip (Infinium 450k). Despite the wealth of tools for Infinium 450k analysis, the identification of the most biologically relevant DNA methylation changes is still challenging. Here we propose an analytical pipeline to select differentially methylated regions (DMRs), tailored on microarray architecture, which is highly effective in highlighting biologically relevant results. The pipeline groups microarray probes on the basis of their localization respect to CpG islands and genic sequences and, depending on probes density, identifies DMRs through a single-probe or a region-centric approach that considers the concomitant variation of multiple adjacent CpG probes. We successfully applied this analytical pipeline on 3 independent Infinium 450k datasets that investigated age-associated changes in blood DNA methylation. We provide a consensus list of genes that systematically vary in DNA methylation levels from 0 to 100 years and that have a potentially relevant role in the aging process.

## INTRODUCTION

In the last two years the Infinium HumanMethylation-450 BeadChip (Infinium 450k) [1] has been largely used to investigate age-associated changes in DNA methylation profile of the human genome [2–9]. The Infinium 450k contains 485577 probes, 64% of them mapping to CpG islands and CpG islands surrounding regions (shores and shelves), while the remaining mapping to dispersed CpG sites in the genome [10]. The array is highly informative, as it covers 96% of the CpG islands of the genome and 99% of RefSeq genes.

Using the Infinium 450k, researchers have identified several CpG sites that either get hypermethylated or hypomethylated during aging in different tissues [11], and a subset of these CpG sites has been successfully combined in predictors of chronological age [12, 13].

Although defining a list of CpG sites whose methylation status is age-dependent is an essential step in aging research, the real challenge is to identify biologically relevant DNA methylation changes and their relative contribution to the aging process.

The difficult task of extracting relevant information from microarray data can be made easier if the number of microarray features is reduced on the basis of a biologically meaningful criterion. In this way the top ranking groups of features are more likely to be functionally linked to the phenotype under study than the single features. For expression microarrays this task has been successfully addressed by grouping genes that share common biological functions [14], but this approach is less suitable for methylation microarrays, as the relationship between DNA methylation and biological function is complex. An alternative solution is to adopt a region-centric approach in which the methylation value not of the single CpG probes, but of a group of adjacent CpG probes is considered. This approach is particularly interesting as changes in DNA methylation, especially in the CpG islands, usually involve groups of adjacent CpG sites whose methylation levels are correlated, thus potentially affecting chromatin structure. On the contrary the biological relevance of alterations at individual CpGs, although potentially interesting at specific genomic regions, is less characterized [15].

At present, different region-centric approaches have been proposed. Illumina Methylation Analyzer (IMA) defines for each gene 11 region categories (TSS1500, TSS200, 5′ UTR, 1^st^ EXON, GENE BODY, 3′ UTR, ILSAND, NSHORE, SSHORE, NSHELF, SSHELF) and calculates their mean or median methylation values, which are then compared between the samples under analysis [16]. As an alternative approach, Numerical Identification of Methylation Biomarker Lists (NIMBL) reports the number of differentially methylated probes within the different annotated regions of each gene [15]. A more sophisticated approach is based on the “bump hunting” method developed for the analysis of CHARM data [17], but its applicability to Infinium 450k data is weakened by the lower density of analysed CpG sites in comparison to CHARM array.

The main point is that the Infinium 450k probes are not evenly distributed across the genome, but they are enriched in specific regions while others are underrepresented. To deal with this issue, a methodology in which differentially methylated regions (DMRs) are defined as regions in which at least two contiguous probes within 1-kb distance have a significant differential statistic was recently proposed [18] and used in a meta-analysis to identify age associated DMRs. A tool for DMRs identification at a region level was implemented also in the RnBeads package, where regions were defined based on the microarray annotation and ranked based on 3 criteria (mean of the differences between average methylation levels of the probes in a region in the two groups under investigation, mean of quotients and a combined *p*-value calculated from the single *p*-values of the probes in the region).

Here we implemented an alternative pipeline for the analysis of Infinium 450k data that is based on a careful description of CpG probes distribution within the array. The proposed methodology: 1) classifies CpG probes based on their genomic localization 2) defines groups of adjacent CpG probes based on their density in the region 3) depending on the previous classifications, applies a single-probe or a region centric analysis which considers the concomitant variation of a group of adjacent CpG probes.

As a proof of principle, we used our approach to conduct a meta-analysis on 3 independent datasets in which the Infinium 450k was used to investigate age-associated variations in blood DNA methylation profiles. From this meta-analysis, we extracted a short list of genes that potentially have a biologically relevant role in the aging process.

## RESULTS

### Grouping Infinium 450k probes in biologically meaningful clusters

**Figure 1:**
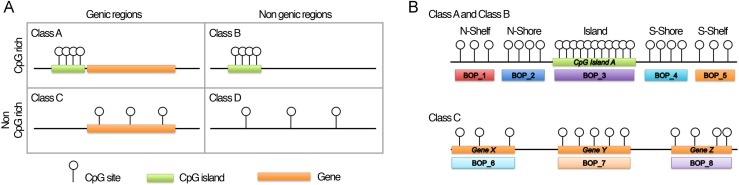
Infinium 450k probes classification and BOPs definition (**A**) The 485577 probes included in the Illumina HumanMethylation450 BeadChip were divided in 4 classes on the basis of their genomic localization. (**B**) Graphic representation of how probes were grouped in BOPs. Probes mapping in the island and in the surrounding regions of the same CpG island were grouped in 5 functional units: probes in the N-Shelf of the island, probes in the N-Shore of the island, probes in the island, probes in the S-Shore of the island, probes in the S-Shelf of the island. Probes mapping in gene bodies were grouped on the basis of the gene in which they are located.

The method we propose focuses on grouping CpG probes into clusters, hereafter referred as “blocks of probes” (BOPs). CpG probes were grouped taking into consideration not only their contiguity in DNA sequence, but also their genomic localization, which represents a critical aspect for data interpretation [19]. Using Illumina probe annotation, we first divided the probes included in the array in four classes (Fig. 1A; see Materials and Methods section): *i)* Class A, including probes in CpG islands and CpG islands-surrounding sequences (shores and shelves) that map in genic regions; *ii)* Class B, including probes in CpG islands and CpG islands-surrounding regions (shores and shelves) which do not map in genic regions; *iii)* Class C, including probes in genic regions which are not CpG rich; *iv)* Class D, including probes in non-genic regions which are not CpG rich. These four classes have different epigenetic functions, as their methylation status can affect gene function and chromatin structure in different ways [20]. Class A, B, C and D included 247394, 62071, 118206 and 57906 CpG probes respectively.

Then, we defined BOPs as follows (Fig. 1B): for probes mapping in CpG islands and in CpG islands surrounding regions (Class A and Class B), we grouped the CpG probes localized in the same island, in the same shore or in the same shelf; for probes mapping in not CpG rich genic regions (Class C), we grouped the CpG probes mapping to the same gene. Class D probes were not grouped because they were highly interspersed across the genome.

**Figure 2:**
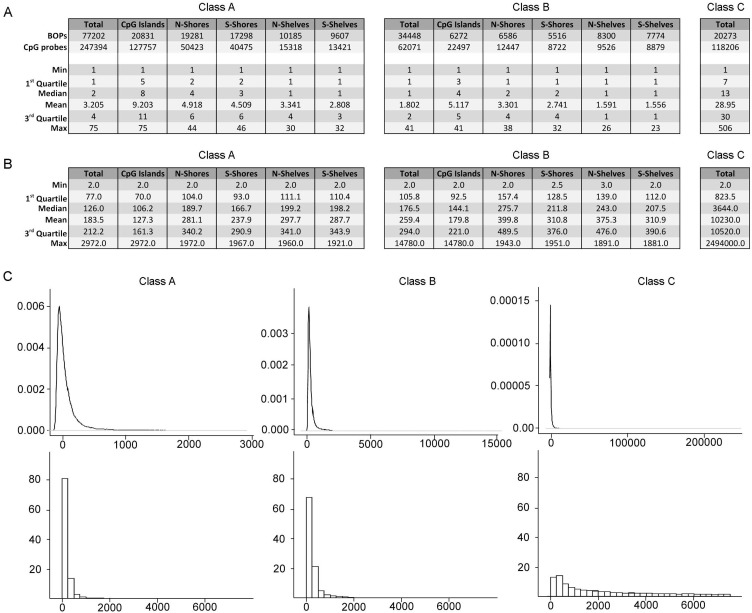
Characteristics of the BOPs belonging to different probe classes (**A**) Numbers of BOPs and CpG probes in Class A, Class B and Class C. For Class A and Class B, subdivision in CpG islands, N-Shores, S-Shores, N-Shelves and S-Shelves is reported. In the lower part of the tables, descriptive statistics for the distribution of number of probes/BOP in the 3 Classes are reported. (**B**) Descriptive statistics for the distribution of mean bp distance between probes /BOP in the 3 Classes are reported.(**C**) Density distributions (upper panel) and frequency histograms (lower panels) of the mean bp distance between the probes/BOP.

Class A, B and C included 77202, 34448 and 20273 BOPs respectively (Fig. 2). Class A BOPs mapped mainly to CpG islands and shores, but CpG islands BOPs were definitely richer in CpG probes (Fig. 2A, left panel). Class B BOPs mapped to shelves with higher frequency than Class A BOPs, but also in this case CpG probes mapped mostly to CpG islands (Fig. 2A, middle panel). As expected Class C BOPs contained a very high number of CpG probes, with a median of 13 and a maximum of 506 (Fig. 2A, right panel).

A region-centric approach is meaningful only if CpG probes are sufficiently close along the DNA sequence under investigation. Indeed, experimental evidences indicate that DNA methylation of nearby CpG sites is correlated within a tract of 250–500 bp [21]. We therefore calculated the mean distance between the probes in each BOP for Class A, Class B and Class C probes (Fig. 2B). Probes in BOPs belonging to Class A and Class B were generally close, with a mean distance of 183.5 and 259.4 bp respectively (Fig. 2B, left and middle panels) and a mode of 73 and 130 bp respectively (Fig. 2C, left and middle panels). In both the cases, as expected, mean distance was lower in islands than in shores and shelves. On the contrary, BOPs belonging to Class C included probes that were scattered across the length of gene sequences and, on average, that were too distant to be analysed together (mean distance 10230 bp, mode 1935 bp; Fig. 2B and 2C, right panels).

### DMR identification by multivariate analysis of variance (MANOVA)

**Figure 3:**
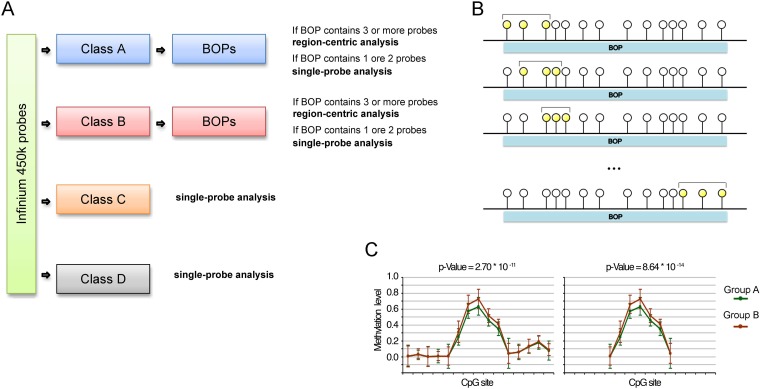
Proposed analytical pipeline for Infinium 450k data (**A**) Workflow for the use of single-probe or region-centric approaches on Infinium 450k data. (**B**) Graphical representation of the sliding window MANOVA used to normalize for BOPs lengths. CpG probes are represented as circles. The CpG probes considered in each round of MANOVA are highlighted in yellow. (**C**) Example of methylation values of CpG probes within a BOP. The BOP includes 22 CpG probes, 5 of which define a “bubble” of differential methylation between Group A and Group B. The *p*-value derived from MANOVA on this BOP is 2.70*10e-11. We hypothesized to have a shorter BOP including only the 5 CpG probes differentially methylated between Group A and Group B, plus a probe on both the sites whose methylation level is comparable between the two samples. In this case, although the extent of the bubble of differentially methylation is the same of the longer BOP, the *p*-value derived from MANOVA is lower, equal to 8.64*10e-14. This simple example shows that if we do not normalize for the length of the BOP, short BOPs tend to rank at higher positions than long BOPs.

Based on the previous observations we propose an analysis pipeline for Infinium 450k “customized” on the characteristics of the different classes of probes (Fig. 3A). BOPs belonging to Class A and Class B are suitable for a region-centric analysis, while for Class C and Class D probes a single-probe analysis is more advisable.

For the region-centric analysis we propose the use of multivariate analysis of variance (MANOVA) to test for general changes in methylation of a genomic region. However, as described above, Class A and Class B BOPs contain a largely variable number of CpGs, spanning from 1 to 75 for Class A BOPs and from 1 to 41 for Class B BOPs. We considered BOPs containing 1 or 2 CpG probes not informative enough for a region-centric approach but more suitable for a single-probe approach. On the contrary, the remaining BOPs (32356 BOPs including 192465 probes in Class A; 7253 BOPs including 31077 probes in Class B) are analysed by applying a sliding-window MANOVA (Fig. 3B). Indeed, the non-homogeneous distribution in BOP probe content could lead to overrepresentation of short BOPs among the top significant BOPs, because when the number of probes in a BOPs is high, it is likely that only a subgroup of them is differentially methylated in the phenotype under study (see the example reported in Fig. 3C). In the sliding window approach we calculated MANOVA for each subgroup of 3 consecutive CpG within the same BOP and we kept the lowest *p*-value among those calculated, actually normalizing the analysis for the varying number of probes per BOP.

We implemented the proposed pipeline in R software environment. Starting from a table containing the beta-values of interest (1 row per CpG site, 1 column per individual), the script annotates the probes and divides them in Class A, Class B, Class B and Class D. Class A and Class B probes are grouped in BOPs and BOPs containing 3 or more probes are analysed by the region-approach (MANOVA on sliding windows of 3 CpG probes within the same BOP). The remaining CpG probes are analysed by ANOVA. Both categorical and continuous covariates can be used. A list of significant CpG probes is outputted from the single-probe analysis. For the region-centric approach, a list of BOPs ranked by nominal or FDR-corrected *p*-values and, if present, associated genes, is provided as output. Then, authors can choose to select significant BOPs containing at least 2 adjacent CpG sites for which the DNA methylation difference between the considered groups (in the case of a categorical variable) or two selected ranges of values of the continuous variable is higher than a set threshold.

Significant BOPs can be ranked according to this criterion allowing authors to identify “bubbles” of different methylation between the conditions under study, which are likely to be biologically meaningful. The pipeline provides as outputs several useful plots, including schematic diagrams in which the chromosomal position of the probes within the selected BOPs is plotted against the methylation level of each probe (see the examples reported in Fig. 6 and in Supplementary Fig. 1). These plots provide a easy-to-interpret visualization of relevant results, that in one shot gives information about probes density within the region and about the changes in DNA methylation between the groups under study.

### Identification of age-associated DMR through the region-centric approach

We validated our pipeline on three independent age-related Infinium 450k experiments performed on whole blood. The first dataset (referred hereafter as D1) includes data from 656 subjects ranging from 19 to 101 years (average 64 ± 15 years) [3]. The second dataset (referred hereafter as D2) includes data from 38 subjects, 19 newborns and 19 nonagenarians [2]. The third dataset (referred hereafter as D3) includes data from 58 subjects ranging from 9 to 83 years (average 44 ± 18 years) [4].

In each dataset, probes containing missing values in at least one sample and probes on X and Y chromosome were removed. Age-associated DMR were identified in D1 and D3 using the age as a continuous variable and in D2 the group (newborns or nonagenarians) as a categorical variable. Ethnicity (Caucasian-European or Hispanic-Mexican) was used as covariate in the D1 analysis. As the relative proportions of the different types of blood cells can vary significantly with age, we inferred blood cell counts from methylation data and use them as covariates in the analysis.

Here we present results only for the region-centric analysis performed on Class A BOPs containing at least 3 CpG probes. MANOVA results were corrected for multiple comparisons through Benjamini-Hochberg False Discovery Rate correction; 0.05 was used as significant threshold for *q*-values. Based on these criteria 21083, 517 and 2736 BOPs were identified as age-associated DMRs in D1, D2 and D3 respectively.

The considerably lower number of BOPs identified in D2 is ascribable to the effect of blood cell types correction, as cord blood and whole blood significantly differ for this parameter.

**Figure 4:**
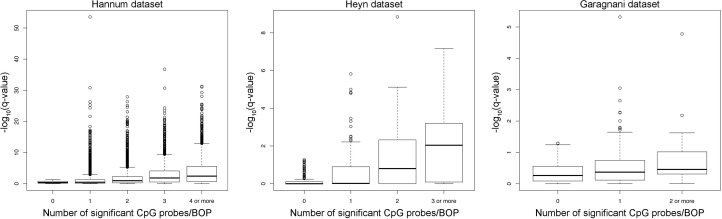
Number of significant CpG probes per significant BOP For each dataset, the boxplot reports the –log_10_(q-value) of each significant Class A BOP (MANOVA analysis) against the number of significant CpG probes (q-value < 0.05, ANOVA analysis) included in each BOP.

To validate our approach, we compared the results of the region-centric approach with the results of a single-probe approach by analysis of variance (ANOVA). As shown in Figure 4, the number of significant CpG probes per BOP tended to be higher in the top ranking BOPs identified by the region-centric analysis. In most the cases, significant CpG probes within the same BOP concordantly moved towards hypermethylation or hypomethylation in younger compared to older subjects. Notably a small number of the selected BOPs in the three datasets included slightly differentially methylated CpG probes that did not reach the significance threshold by themselves, but whose concomitant variation with adjacent probes within the same BOP resulted significant when analysed by a multivariate approach. These results indicate that our approach was successful in identifying chromosomal regions, rather than single CpG sites, whose methylation status is affected by aging.

To confirm that our region-centric approach reduces spurious results and is more likely to identify biologically relevant regions, we compared the results from the three datasets.

**Figure 5:**
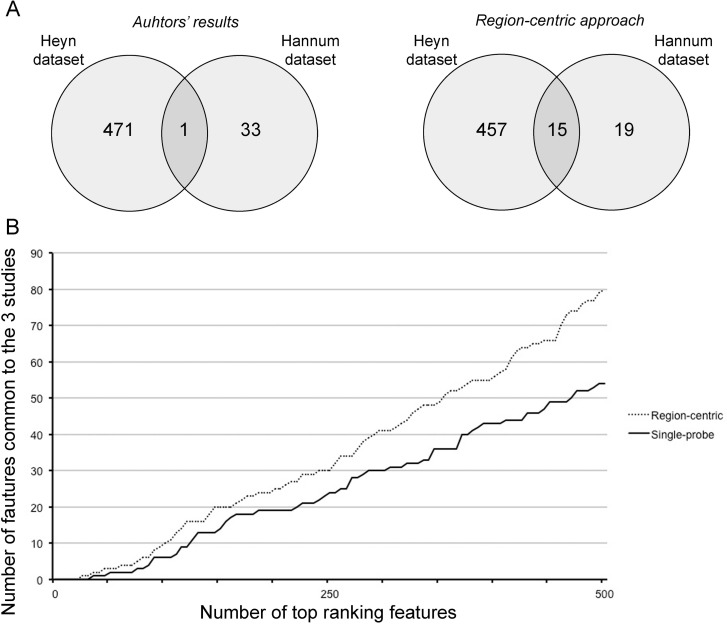
The region-centric approach increases the common findings between the 3 datasets (**A**) Intersection between the results provided by Hannum et al. and Heyn et al. (left panel) and between the results of the region-centric approach on the two datasets. (**B**) Intersection between a progressively increasing number of top ranking features (BOPs for the region-centric analysis, CpG probes for the single-probe analysis) in the three datasets.

First, we compared the DMRs identified in Hannum et al. and in Heyn et al. with those identified by our approach. Hannum et al. used a multivariate linear model approach based on the Elastic Net algorithm and identified a nucleus of 89 CpG probes whose methylation is associated to age, while Heyn et al selected 3205 age-associated CpG probes that resulted significant after ANOVA test (*q*-value < 0.01) and that showed a difference in average beta-values between newborns and nonagenarians greater than 0.20. We considered only the CpG probes belonging to Class A probes, that is 45 and 800 probes in D1 and D2 respectively, and we matched them with the corresponding BOPs. In this way, we achieved a list of 34 and 472 BOPs identified by Hannum et al. and by Heyn et al.. Only one BOP was shared by the two lists (Fig 5A, left panel). On the contrary, when we considered the first 34 and the first 472 BOPs identified by our approach respectively in D1 and D2, we observed an overlap of 15 BOPs (Fig 5A, right panel).

Secondly, we considered a progressively increasing number of significant BOPs (region-centric analysis) and CpG probes (single-probe analysis) and we calculated the number of common DMRs between the three datasets. As shown in Figure 5B, the extent of overlap was higher for the region-centric approach compared to the single-probe approach.

**Figure 6:**
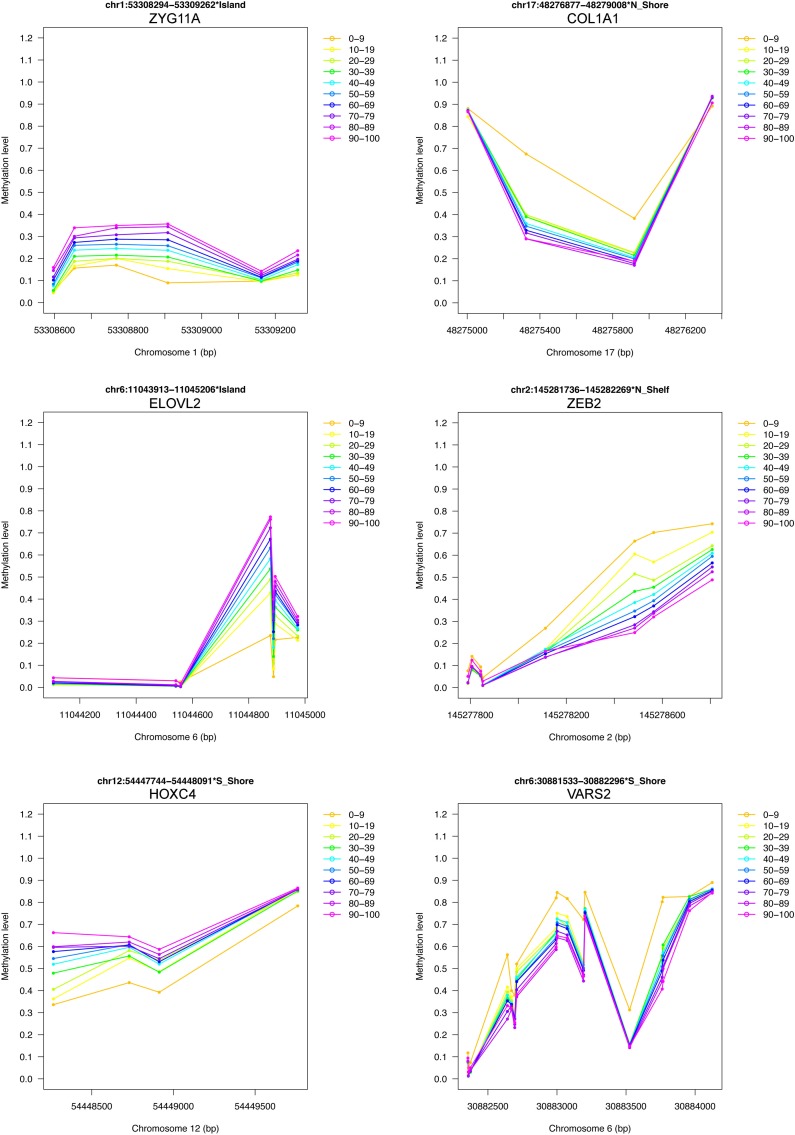
Examples of DNA methylation profiles of selected age-associated BOPs 6 of the 42 selected BOPs are reported as an example. Mean methylation values in 10 age classes are reported for each CpG probe within the selected BOPs. For each BOP, beta-values from D1, D2 and D3 were joined together.

Finally, we provided a short list of genomic regions whose methylation levels vary according to age in D1, D2 and D3 (Table 1). We considered the overlap between the first 500 ranked BOPs identified by our region-centric approach in the 3 datasets, resulting in 42 BOPs (44 genes, because some BOPs mapped to multiple genes which share the same CpG island). 11 and 2 of the selected BOPs included at least one CpG probe that was provided also in Hannum's and Heyn's results respectively. Moreover, 4 of the selected BOPs contained at least one CpG probe that was included also in Horvath's epigenetic clock. Two genes (HOXC4 and SST) were included in the GenAge database as related to aging in model systems and/or humans [22]. To have a general view of age-associated changes of the selected BOPs, we joined their beta-values from D1, D2 and D3 and we divided samples in 10 age ranges from 0 to 100 years. The plots reported in Fig 6 and in Supplementary Figure 1 confirm that the 42 genomic regions encounter a systematic hypermethylation (20 BOPs) or hypo-methylation (22 BOPs) with age.

**Table 1:** Candidate age-associated genomic regions

Gene Name	Description	BOP	Hannum	Heyn	Horvath
*ABCC4*	ATP-binding cassette, sub-family C (CFTR/MRP), member 4	chr13:95953337-95954211*N_Shore			
*ABHD14A*	abhydrolase domain containing 14A	chr3:52008943-52009339*N_Shore	X		X
*ABHD14B*	abhydrolase domain containing 14B	chr3:52008943-52009339*N_Shore	X		X
*AKAP8L*	A kinase (PRKA) anchor protein 8-like	chr19:15529290-15529902*S_Shore			
*ALDOA*	aldolase A, fructose-bisphosphate	chr16:30076310-30077872*N_Shore			
*AMER3*	APC membrane recruitment protein 3	chr2:131513363-131514183*Island	X		
*ATP13A2*	ATPase type 13A2	chr1:17337829-17338590*S_Shore	X		
*AXL*	AXL receptor tyrosine kinase	chr19:41769215-41769417*N_Shore	X		
*CACNA1G*	calcium channel, voltage-dependent, T type, alpha 1G subunit	chr17:48636103-48639279*Island			
*COL1A1*	collagen, type I, alpha 1	chr17:48276877-48279008*N_Shore			
*CPEB1*	cytoplasmic polyadenylation element binding protein 1	chr15:83315116-83317541*Island		X	
*CSNK1D*	casein kinase 1, delta	chr17:80231019-80231820*S_Shore			X
*EDARADD*	EDAR-associated death domain	chr1:236558459-236559336*N_Shore	X		X
*EIF1*	eukaryotic translation initiation factor 1	chr17:39844833-39845950*N_Shore	X		
*ELOVL2*	ELOVL fatty acid elongase 2	chr6:11043913-11045206*Island	X		
*FHL2*	four and a half LIM domains 2	chr2:106014878-106015884*Island	X	X	
*GIT1*	G protein-coupled receptor kinase interacting ArfGAP 1	chr17:27918161-27918398*N_Shore			
*GLRA1*	glycine receptor, alpha 1	chr5:151304226-151304824*Island			
*GPR78*	G protein-coupled receptor 78	chr4:8582036-8583364*Island			
*GRIN2C*	glutamate receptor, ionotropic, N-methyl D-aspartate 2C	chr17:72848166-72848901*Island			
*GUSB*	glucuronidase, beta	chr7:65446771-65447340*S_Shore			
*HNRNPUL1*	heterogeneous nuclear ribonucleoprotein U-like 1	chr19:41769215-41769417*N_Shore	X		
*HOXC4*	homeobox C4	chr12:54447744-54448091*S_Shore	X		
*IRX5*	iroquois homeobox 5	chr16:54962422-54967805*Island			
*LAG3*	lymphocyte-activation gene 3	chr12:6882855-6883184*N_Shore			X
*MLXIPL*	MLX interacting protein-like	chr7:73037528-73038957*Island			
*NENF*	neudesin neurotrophic factor	chr1:212606105-212606844*N_Shore			
*NFIA*	nuclear factor I/A	chr1:61548753-61549564*N_Shore			
*OTUD7A*	OTU deubiquitinase 7A	chr15:31775540-31776988*Island	X		
*PI4KB*	phosphatidylinositol 4-kinase, catalytic, beta	chr1:151300522-151300724*N_Shore			
*PRLHR*	prolactin releasing hormone receptor	chr10:120353692-120355821*Island			
*PRRT4*	proline-rich transmembrane protein 4	chr7:127990926-127992616*Island			
*PTGDS*	prostaglandin D2 synthase 21kDa (brain)	chr9:139872237-139873143*N_Shore			
*PXN*	paxillin	chr12:120702976-120703541*S_Shore			
*RCSD1*	RCSD domain containing 1	chr1:167599464-167599839*N_Shore			
*SLC12A5*	solute carrier family 12 (potassium/chloride transporter), member 5	chr20:44657463-44659243*Island			
*SLC25A22*	solute carrier family 25 (mitochondrial carrier: glutamate), member 22	chr11:797640-798544*N_Shore			
*SOX1*	SRY (sex determining region Y)-box 1	chr13:112720564-112723582*Island			
*SST*	somatostatin	chr3:187387914-187388176*N_Shore	X		
*TFAP2B*	transcription factor AP-2 beta (activating enhancer binding protein 2 beta)	chr6:50787286-50788091*Island			
*VARS2*	valyl-tRNA synthetase 2, mitochondrial	chr6:30881533-30882296*S_Shore			
*ZAR1*	zygote arrest 1	chr4:48492117-48493589*Island			
*ZEB2*	zinc finger E-box binding homeobox 2	chr2:145281736-145282269*N_Shelf			
*ZYG11A*	zyg-11 family member A, cell cycle regulator	chr1:53308294-53309262*Island			

## DISCUSSION

In this paper we present an original pipeline for the analysis of Infinium 450k data, in which different genomic regions are analyzed either by a single-probe or a region-centric approach depending on their context and probe content/density.

The Infinium 450k is currently the most used technology for EWAS studies. The reasons for the success of this microarray are to be found in its affordability and simplicity, in addition to a reasonable informativeness on genome-wide DNA methylation profiles. Alternative techniques such as sequencing of immunoprecipitated methylated DNA (MeDIP-seq) or of bisulfite-treated genomic DNA (BS-seq) provide a more comprehensive picture of DNA methylome [23] but, despite the recent introduction of pipelines for their automatization and analysis [24–27], are still more laborious and expensive.

A recent comprehensive review has collected the bioinformatic tools developed to specifically analyze Infinium 450k data [27]. Apart from methods to normalize data and adjust them for technical bias, several algorithms to identify differentially methylated regions (DMRs) between groups of interest have been developed. A growing body of literature suggests to analyze Infinium 450k data by a region-centric approach, with several advantages respect to single-probe analysis [15, 18]. First of all, it better resembles the biological basis of the process, as concordant changes in a group of adjacent CpG sites are more likely to affect the phenotype compared to alterations of single CpG sites. Moreover, a region-centric approach simplifies the identification of genomic regions of interest as provides a shorter list of ranked results and is less prone to provide spurious results due for example to the presence of SNPs in the probes included in the array [18].

Here we reasoned that the statistical analysis of the Infinium 450k microarray should take into account the specific architecture of the microarray. Indeed, we clearly showed that different genomic regions present different level of coverage in terms of probes distribution. We grouped probes in blocks (BOPs) based on microarray annotations and verified that only in CpG islands and in the surrounding regions (shores and shelves) the mean distance between probes in a BOP was within 500 bp, a range in which the methylation values of CpG sites are usually correlated [21]. On the contrary, the density of probes mapping in not CpG-rich regions (not CpG-rich promoters, gene bodies, intergenic regions) was strikingly lower. This means that different regions of the array are more suitable to a single-probe or a region-centric analysis. Noteworthy, the proposed probes classification does not have only a mere methodological value, but it has important biological implications. First of all, the region-centric approach is selectively applied to short regions (most CpG islands are within 3000 bp, while shores and shelves are by definition 2000 bp long), in which the methylation level of CpG probes is more likely to be correlated. Moreover, a growing body of evidences indicates that the function of DNA methylation greatly varies with genomic context [28]. This means for example that methylation of CpG islands, of shores or of gene bodies can differently affect gene expression and that it can be differently affected by the condition under study. Also, the methylation status of non-genic regions can have important consequences, for example by influencing chromatin architecture and stability, and it is likely that the effect of methylation at non-genic CpG islands or at open-sea CpG sites can be different. Analyzing separately these regions that have different functional meaning can therefore facilitate the identification of informative variations in methylation profiles in the model under consideration. Moreover, this approach provides shorter ranked lists of results that can be more easily examined by the researcher.

We used multivariate ANOVA (MANOVA) to test for general changes in methylation in the region-centric analysis. This approach has been previously adopted for the analysis of methylation data [29, 30] as it allows to explore simultaneously the relationship between several dependent variables (in our case, 3 adjacent CpG probes within a BOP) and the independent variables under study. Notably, most of the other algorithms for DMRs identification is not based on a multivariate approach, but combines the results from univariate analyses on adjacent CpG probes [27]. As shown in the meta-analysis, MANOVA can identify not only regions in which multiple adjacent probes are significantly different between the samples, but also regions in which there are concomitant little variations of adjacent probes, none of which would reach the significance threshold by itself in a univariate test. This could be particularly valuable when small differences exist between the samples under study.

Overall the strength of our approach is that not only microarray features are grouped in biologically meaningful groups, but also that the ranking criterion is based on a multivariate approach. Additionally, significant BOPs can be ranked also on the basis of the “bubble” of differential methylation, defined as at least two adjacent probes whose mean methylation in the groups under investigation differs of at least a minimum value. As a confirm of the validity of the approach we demonstrated that, when considering the top ranking BOPs, our method increased the number of common genes identified in all three studies compared to a single-probe analysis, indicating that it is likely to provide a more informative overview of biologically relevant results.

The analysis pipeline we propose is implemented in R software environment and is therefore freely available. Researchers just need to define the analysis parameters, such as the covariates to be used in the ANOVA/MANOVA, the FDR correction method, the significance threshold and the minimum difference between mean methylation values between the groups under investigation.

We used our analysis pipeline to perform a meta-analysis on 3 Infinium 450k datasets that investigated age-associated changes in DNA methylation. The 3 datasets are considerably different in terms of both samples number and age range. Nevertheless, we were able to identify a core of genomic regions whose methylation profiles systematically vary with aging, from newborns to nonagenarians. To our knowledge, this is the first report describing a relatively large number of genomic regions with such characteristics. Only a subset of these genes was identified by Hannum *et al*. and by Heyn *et al*. or was included in Horvath's epigenetic clock. Inferring how the methylation of these regions can contribute to the aging process is out of the scope of this paper, although the life-long variations in DNA methylation that we described are suggestive of a profound link between development and aging [31]. Many genes showed marked differences between cord blood and the other age ranges. Although these differences could in principle be ascribed to differences in blood cells composition, it is tempting to suggest that during the first phases of growth a profound epigenetic remodeling occurs. It is interesting to note that 2 genes from our list (SST and HOXC4) are enclosed as age-related genes in the GenAge database [22]. Moreover, a Pubmed search using the query “gene name AND aging [title/abstract]” gave some interesting hints, as the expression of CACNA1G, COL1A1, LAG3, PTGDS and ZEB2 genes resulted modulated by age in several models [32–38]. COL1A1 and PTGDS emerged from the same study [32] in which hippocampal gene expression in senescent female mice was assessed after long-term exercise. The observation that age-dependent expression of the above mentioned genes was detected in tissues other than blood prompts further studies to evaluate general rearrangements in epigenetic landscapes of different cell types. Finally, the methylation status of ELOVL2, FHL2 and EDARADD genomic regions was previously described as associated to aging [4, 39].

Collectively the above observations indicate that this short list of genes, selected by means of an analytical pipeline that is tailored on the architecture of the microarray and that is more likely to provide biologically relevant findings, can be used as the basis for deeper investigations to shed light on the molecular basis of the aging process.

## METHODS

### Datasets

D1 and D2 are publicly available at NCBI Gene Expression Omnibus (GEO) (http://www.ncbi.nlm.nih.gov/geo/) under accession numbers GSE40279 and GSE30870 respectively. D3 includes 32 mother–offspring couples and is part of a larger datasets submitted to GEO with accession number GSE52588.

### Estimation of cell counts

Abundance measures of blood cell types were estimated using the appropriate option of the DNA Methylation Age Calculator, freely available at https://dnamage.genetics.ucla.edu/ [12], which is in part based on a previously published algorithm [40]. As suggested by authors, methylation measures were corrected for “CD8.naive” (Naive CD8 T cells), “CD8pCD28nCD45RAn” (memory and effector T cells), “PlasmaBlast” (plasmablasts), CD4 T cells, monocytes, granulocytes and natural killer cells.

### BOPs definition and DMR identification

The analytical pipeline is implemented as an R script freely available at https://immunologyomics.unibo.it/labkey/–450K_pipeline.url. For ease of use, the pipeline is split in 3 step: 1) Definition of Classes and BOPs; 2) DMRs identification 3) FDR correction, selection of significant DMRs/probes, sorting of probes and plots. Step 2 and step 3 are separately provided for Class A BOPs containing 3 or more probes, Class A BOPs containing 1 or 2 probes, Class B BOPs containing 3 or more probes, Class B BOPs containing 1 or 2 probes, Class C probes and Class D probes. Detailed explanations of input and output files are provided. A general description of the 3 analytical steps is provided below:

Step 1: Probes containing missing values and probes with a detection *p*-value greater than 0.05 in more than 75% samples were removed, together with those localized on sexual chromosomes. Probes that contained SNPs were annotated as previously described [21]. For probes classification and BOPs definition, the RELATION_TO_UCSC_CPG_ISLAND and the UCSC_REFGEN_NAME columns in the Illumina Infinium 450k annotation were used to subset the array probes in four classes and to group probes in BOPs.

Step 2: DMRs identification is based on MANOVA and ANOVA functions from the R package *car*. For sliding windows MANOVA, the function is applied on sliding windows of 3 consecutive CpGs within the same BOP. For each BOP, the lowest *p*-value among those calculated for the different sliding windows is kept. Both MANOVA and ANOVA analysis support the use of both categorical and continuous covariates. Parallel processing can be used if the computational environment supports it.

Step 3: The analytic pipeline allows to correct MANOVA/ANOVA *p*-values for multiple testing using the correction methods implemented in the R package *multtest*. BOPs/probes can be selected on the basis of a significance threshold on either nominal or FDR-corrected *p*-values. In the case of BOP analysis, BOPs can be ranked on the basis of a user-defined minimum mean methylation difference between adjacent CpG probes. MDS plots, heatmap plots and line plots are generated as outputs.

## SUPPLEMENTARY FIGURES


