# Erratum: A meta-analysis on age-associated changes in blood DNA methylation: results from an original analysis pipeline for Infinium 450k data

**DOI:** 10.18632/aging.100915

**Published:** 2016-04-21

**Authors:** Maria Giulia Bacalini, Alessio Boattini, Davide Gentilini, Enrico Giampieri, Chiara Pirazzini, Cristina Giuliani, Elisa Fontanesi, Daniel Remondini, Miriam Capri, Alberto Del Rio, Donata Luiselli, Giovanni Vitale, Daniela Mari, Gastone Castellani, Anna Maria Di Blasio, Stefano Salvioli, Claudio Franceschi, Paolo Garagnani

**Aging (Albany NY) 2015; 7(2)**: **97-109**.

PMCID: PMC4359692 PMID: 25701668

In this Article, the error was found in Figure 5: http://www.impactaging.com/papers/v7/n2/pdf/100718.pdf.

The correct Figure is provided here.

**Figure 5:**
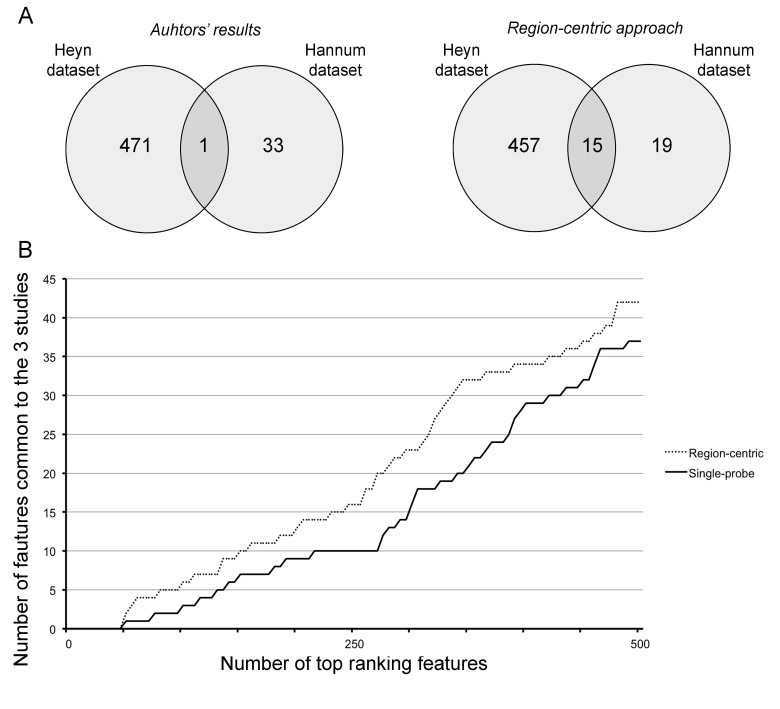
The region-centric approach increases the common findings between the 3 datasets (**A**) Intersection between the results provided by Hannum et al. and Heyn et al. (left panel) and between the results of the region-centric approach on the two datasets. (**B**) Intersection between a progressively increasing number of top ranking features (BOPs for the region-centric analysis, CpG probes for the single-probe analysis) in the three datasets.

