# Supplementary material for: A meta-analysis on age-associated changes in blood DNA methylation: results from an original analysis pipeline for Infinium 450k data
Source: Aging (Albany NY). 2015 Jan 11;7(2):97–109. doi: 10.18632/aging.100718 (PMC4359692; doi:10.18632/aging.100718)
Supplement: Supplementary file 1 [file aging-07-0097-s001.pdf]

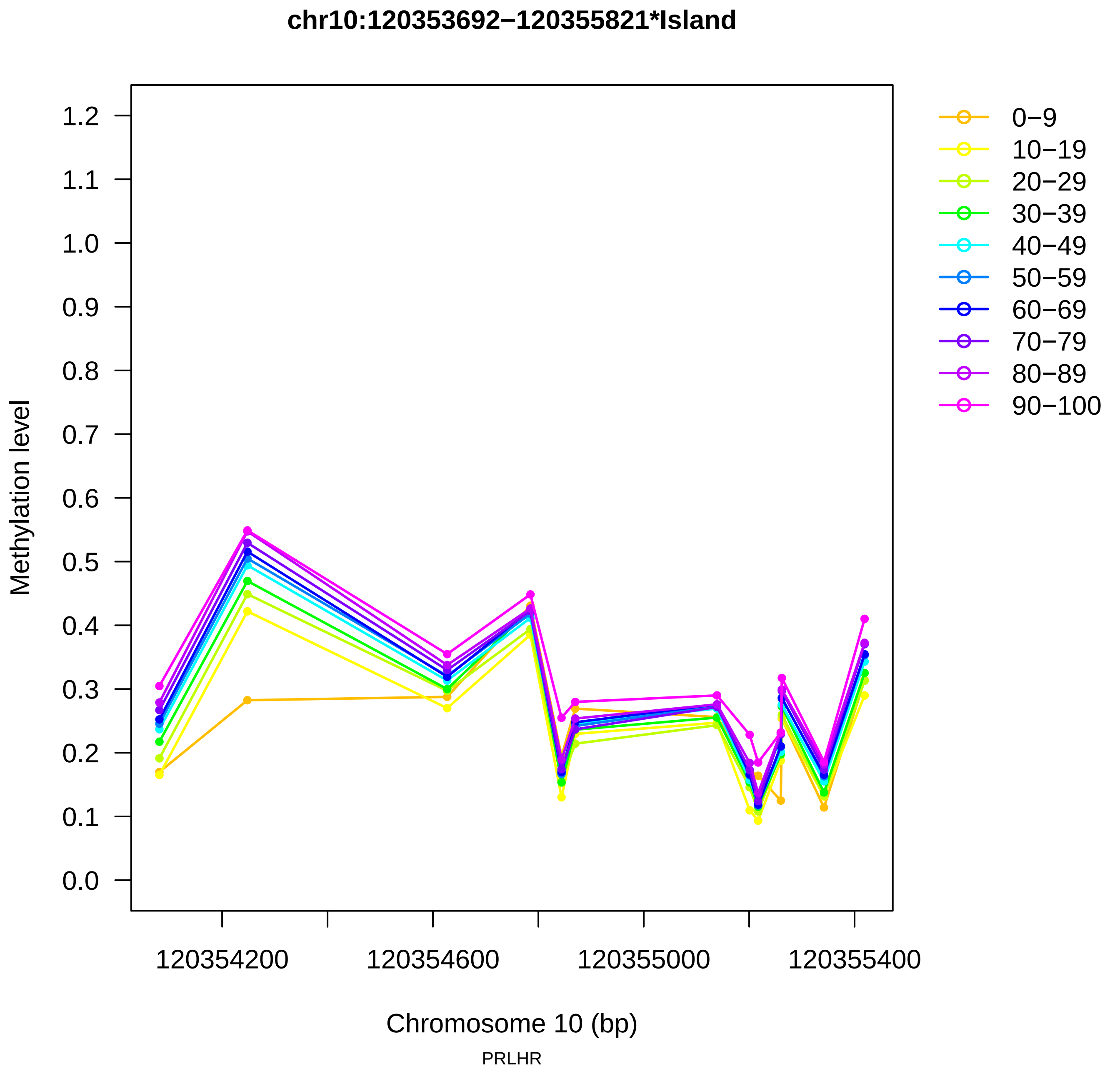
chr10:120353692−120355821*Island
Methylation level
0.0
0.1
0.2
0.3
0.4
0.5
0.6
0.7
0.8
0.9
1.0
1.1
1.2
120354200
120354600
120355000
120355400
Chromosome 10 (bp)
PRLHR
0−9
10−19
20−29
30−39
40−49
50−59
60−69
70−79
80−89
90−100

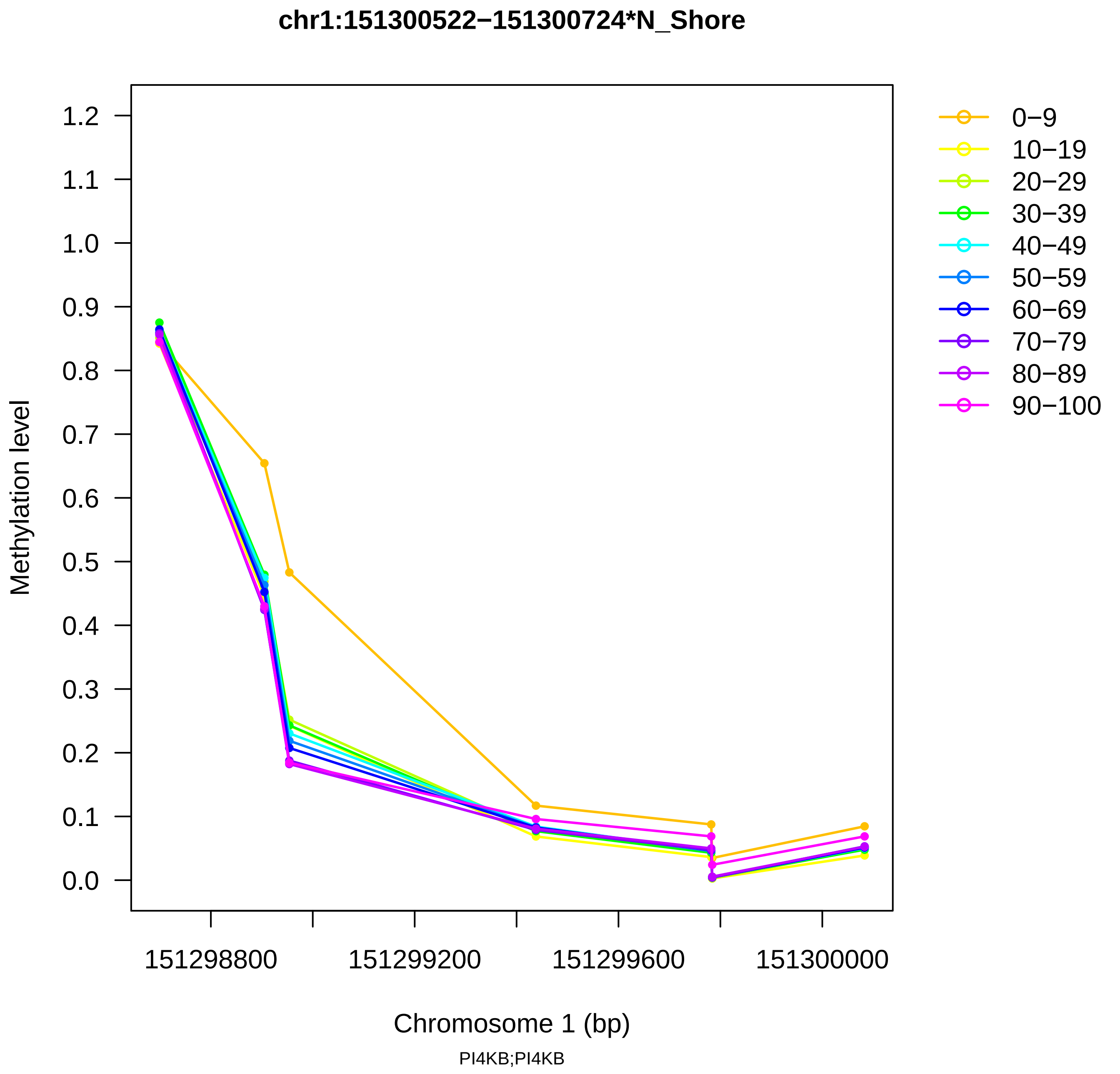
chr1:151300522−151300724*N_Shore
Methylation level
1.2
1.1
1.0
0.9
0.8
0.7
0.6
0.5
0.4
0.3
0.2
0.1
0.0
151298800
151299200
151299600
151300000
Chromosome 1 (bp)
PI4KB;PI4KB
0−9
10−19
20−29
30−39
40−49
50−59
60−69
70−79
80−89
90−100

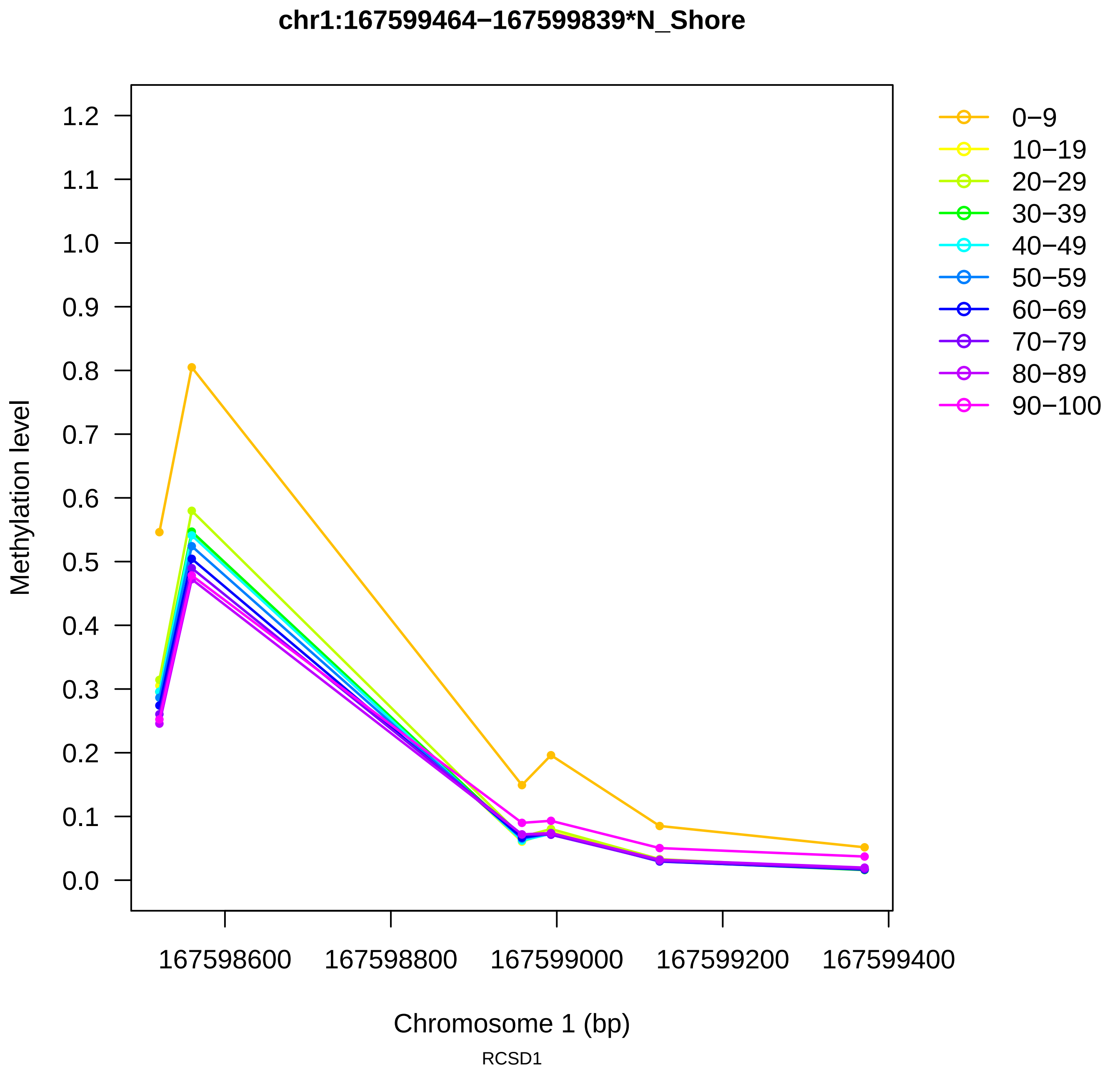
chr1:167599464−167599839*N_Shore
Methylation level
0.0
0.1
0.2
0.3
0.4
0.5
0.6
0.7
0.8
0.9
1.0
1.1
1.2
167598600
167598800
167599000
167599200
167599400
Chromosome 1 (bp)
RCSD1
0−9
10−19
20−29
30−39
40−49
50−59
60−69
70−79
80−89
90−100

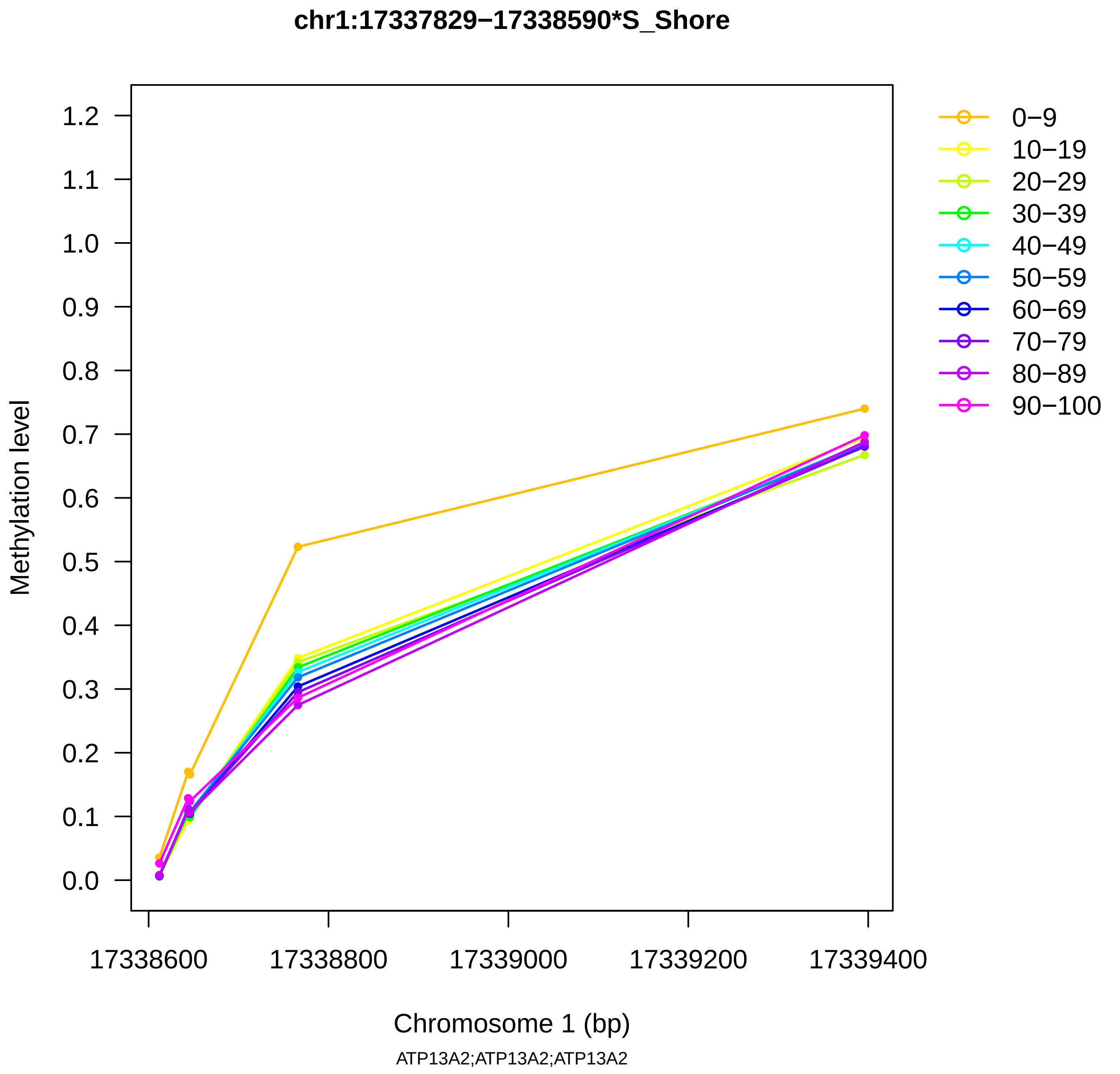
chr1:17337829−17338590*S_Shore
Methylation level
0.0
0.1
0.2
0.3
0.4
0.5
0.6
0.7
0.8
0.9
1.0
1.1
1.2
17338600
17338800
17339000
17339200
17339400
Chromosome 1 (bp)
ATP13A2;ATP13A2;ATP13A2
0−9
10−19
20−29
30−39
40−49
50−59
60−69
70−79
80−89
90−100

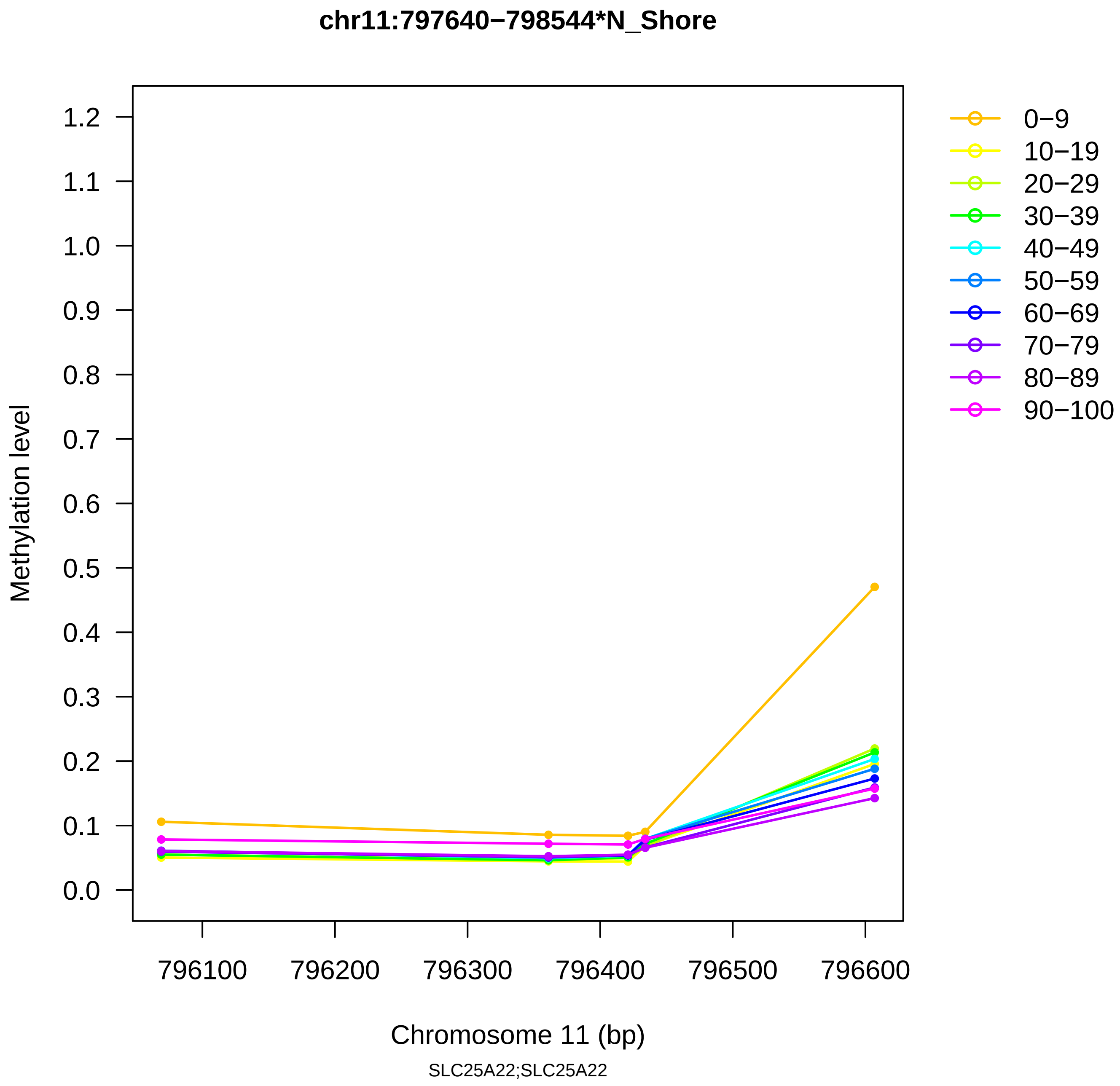

chr11:797640−798544*N_Shore
Methylation level
1.2
1.1
1.0
0.9
0.8
0.7
0.6
0.5
0.4
0.3
0.2
0.1
0.0
796100
796200
796300
796400
796500
796600
Chromosome 11 (bp)
SLC25A22;SLC25A22
0−9
10−19
20−29
30−39
40−49
50−59
60−69
70−79
80−89
90−100

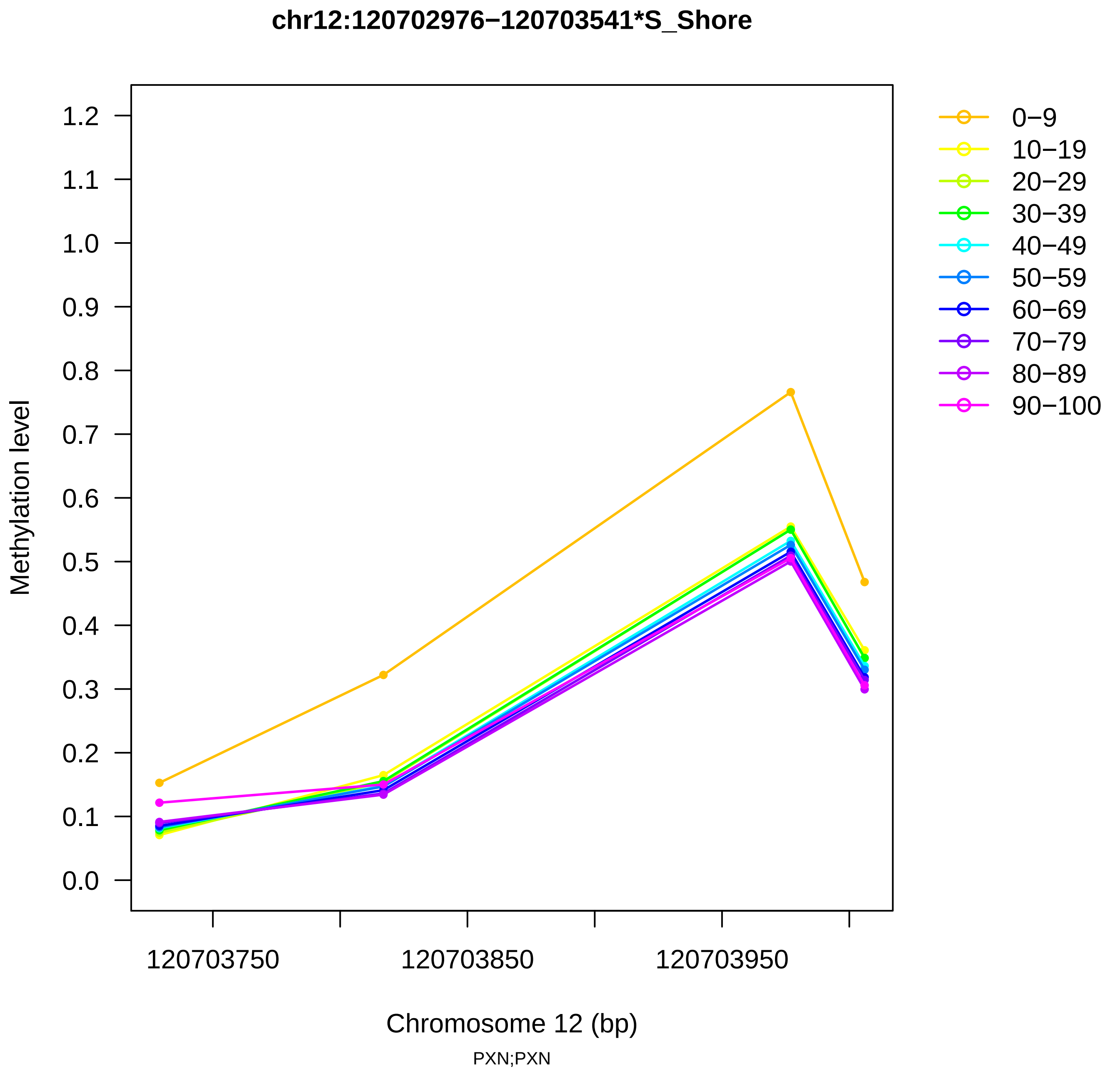

chr12:120702976−120703541*S_Shore
Methylation level
1.2
1.1
1.0
0.9
0.8
0.7
0.6
0.5
0.4
0.3
0.2
0.1
0.0
120703750
120703850
120703950
Chromosome 12 (bp)
PXN;PXN
0−9
10−19
20−29
30−39
40−49
50−59
60−69
70−79
80−89
90−100

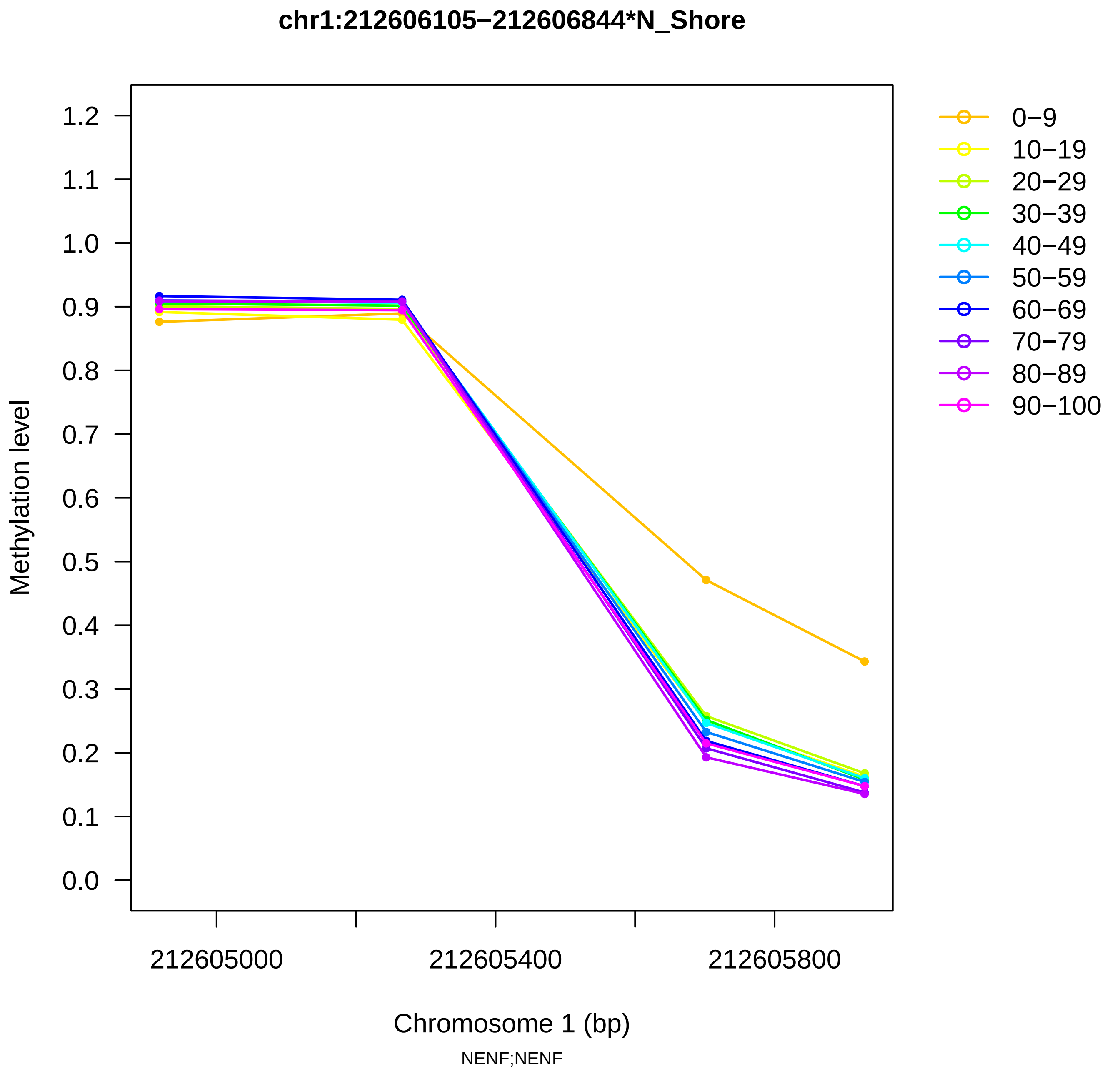
chr1:212606105−212606844*N_Shore
Methylation level
1.2
1.1
1.0
0.9
0.8
0.7
0.6
0.5
0.4
0.3
0.2
0.1
0.0
212605000
212605400
212605800
Chromosome 1 (bp)
NENF;NENF
0−9
10−19
20−29
30−39
40−49
50−59
60−69
70−79
80−89
90−100

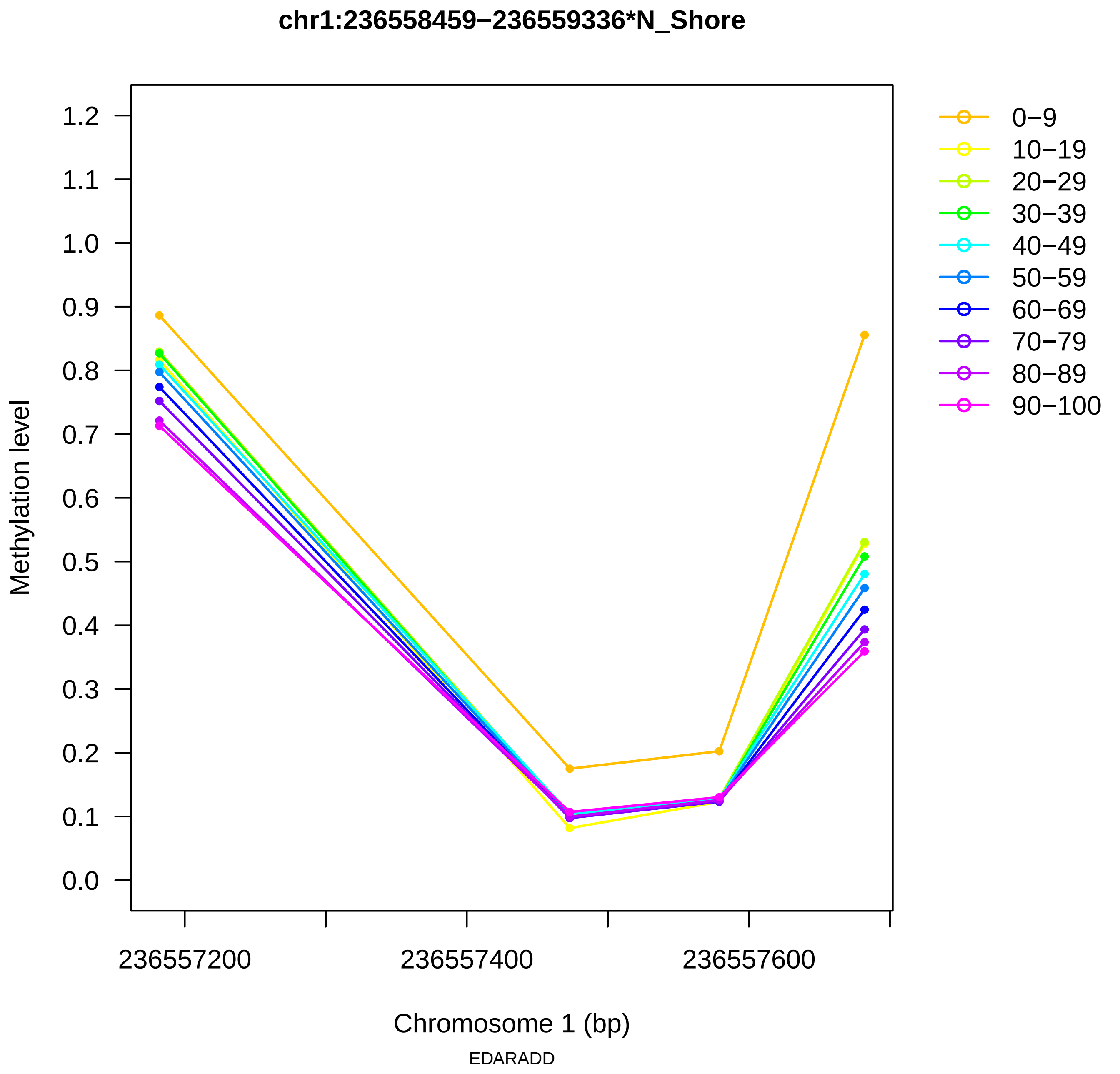
chr1:236558459−236559336*N_Shore
Methylation level
1.2
1.1
1.0
0.9
0.8
0.7
0.6
0.5
0.4
0.3
0.2
0.1
0.0
236557200
236557400
236557600
Chromosome 1 (bp)
EDARADD
0−9
10−19
20−29
30−39
40−49
50−59
60−69
70−79
80−89
90−100

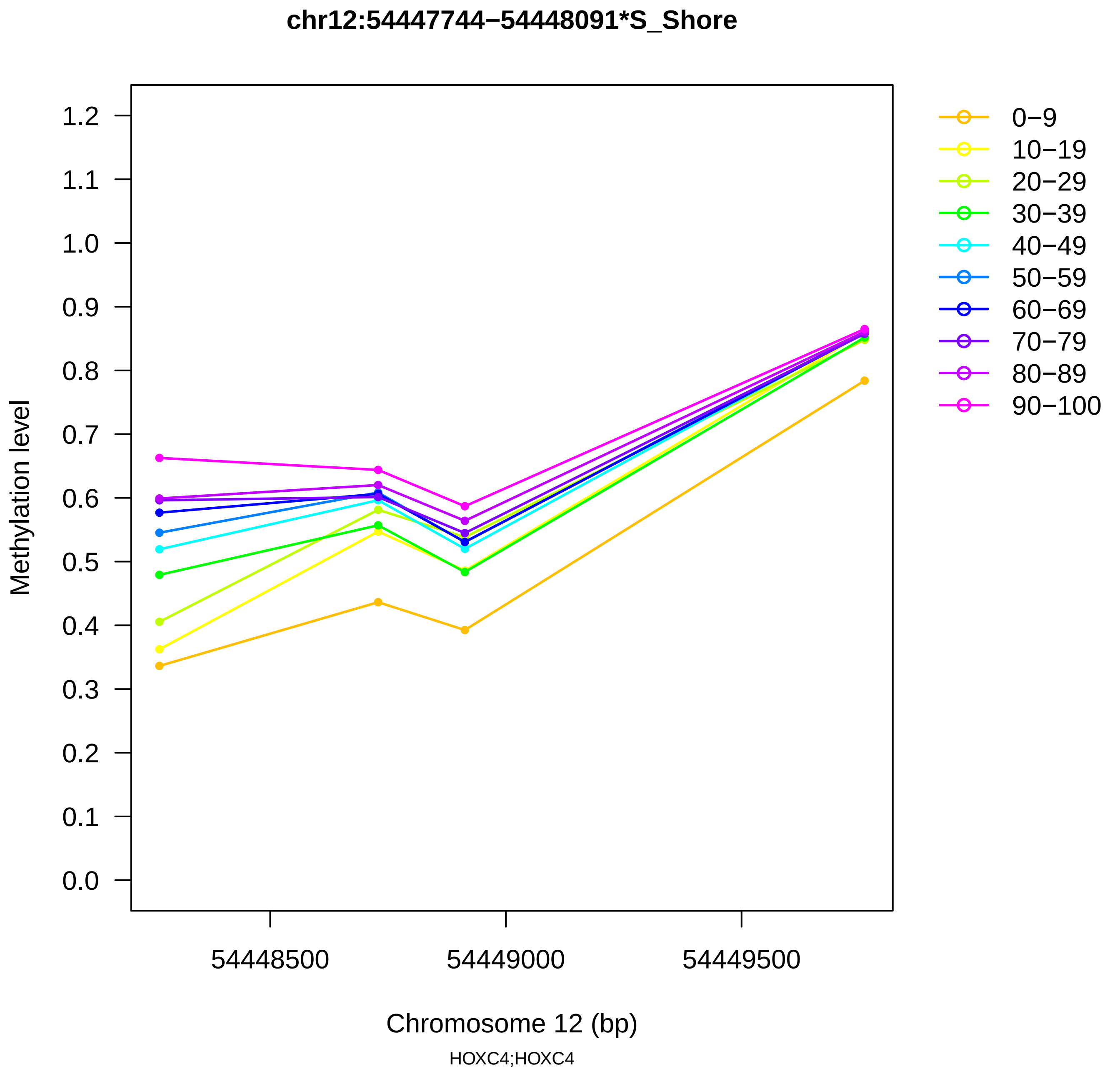

chr12:54447744−54448091*S_Shore
Methylation level
1.2
1.1
1.0
0.9
0.8
0.7
0.6
0.5
0.4
0.3
0.2
0.1
0.0
54448500
54449000
54449500
Chromosome 12 (bp)
HOXC4;HOXC4
0−9
10−19
20−29
30−39
40−49
50−59
60−69
70−79
80−89
90−100

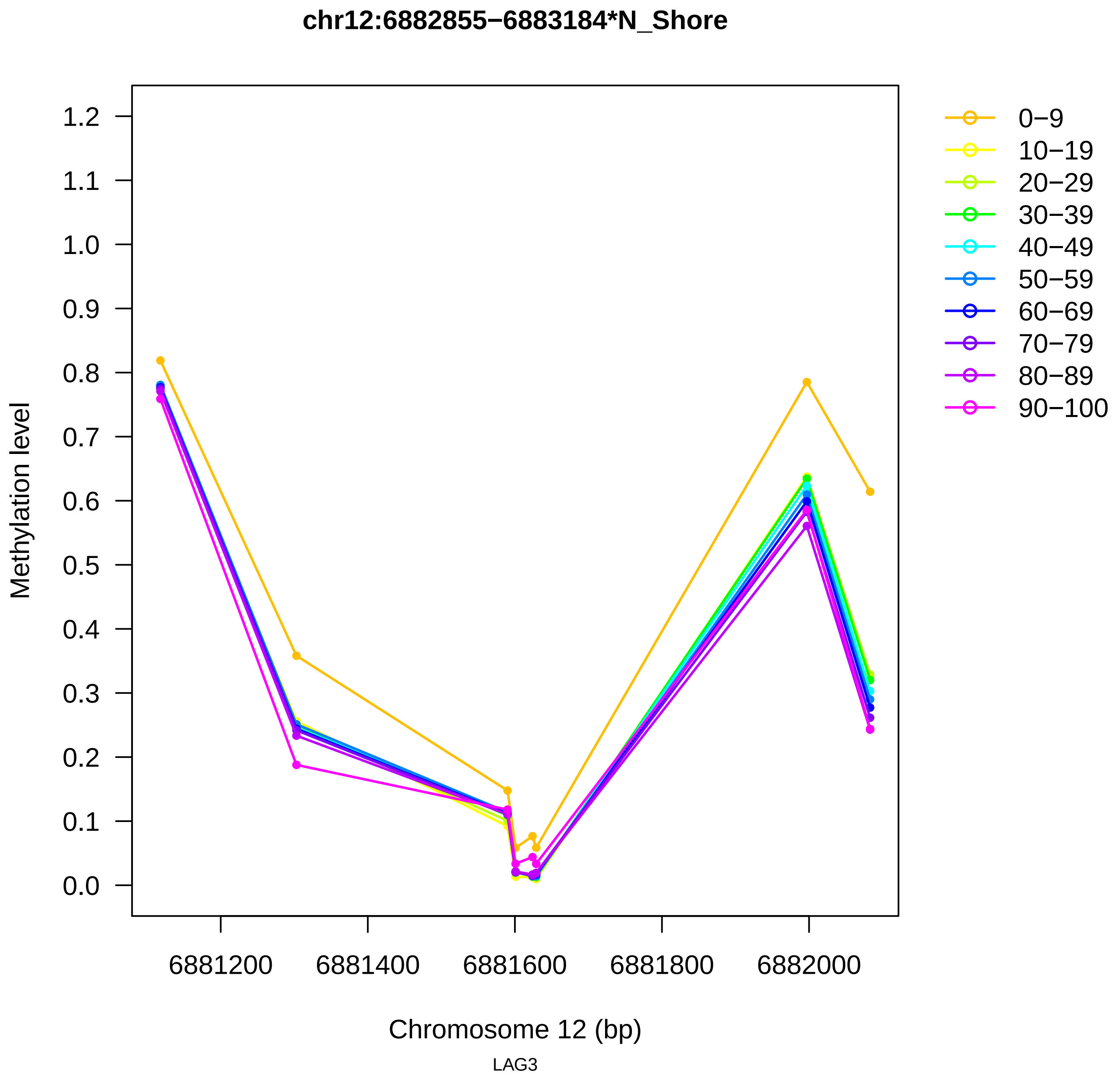
chr12:6882855−6883184*N_Shore
Methylation level
1.2
1.1
1.0
0.9
0.8
0.7
0.6
0.5
0.4
0.3
0.2
0.1
0.0
6881200
6881400
6881600
6881800
6882000
Chromosome 12 (bp)
LAG3
0−9
10−19
20−29
30−39
40−49
50−59
60−69
70−79
80−89
90−100

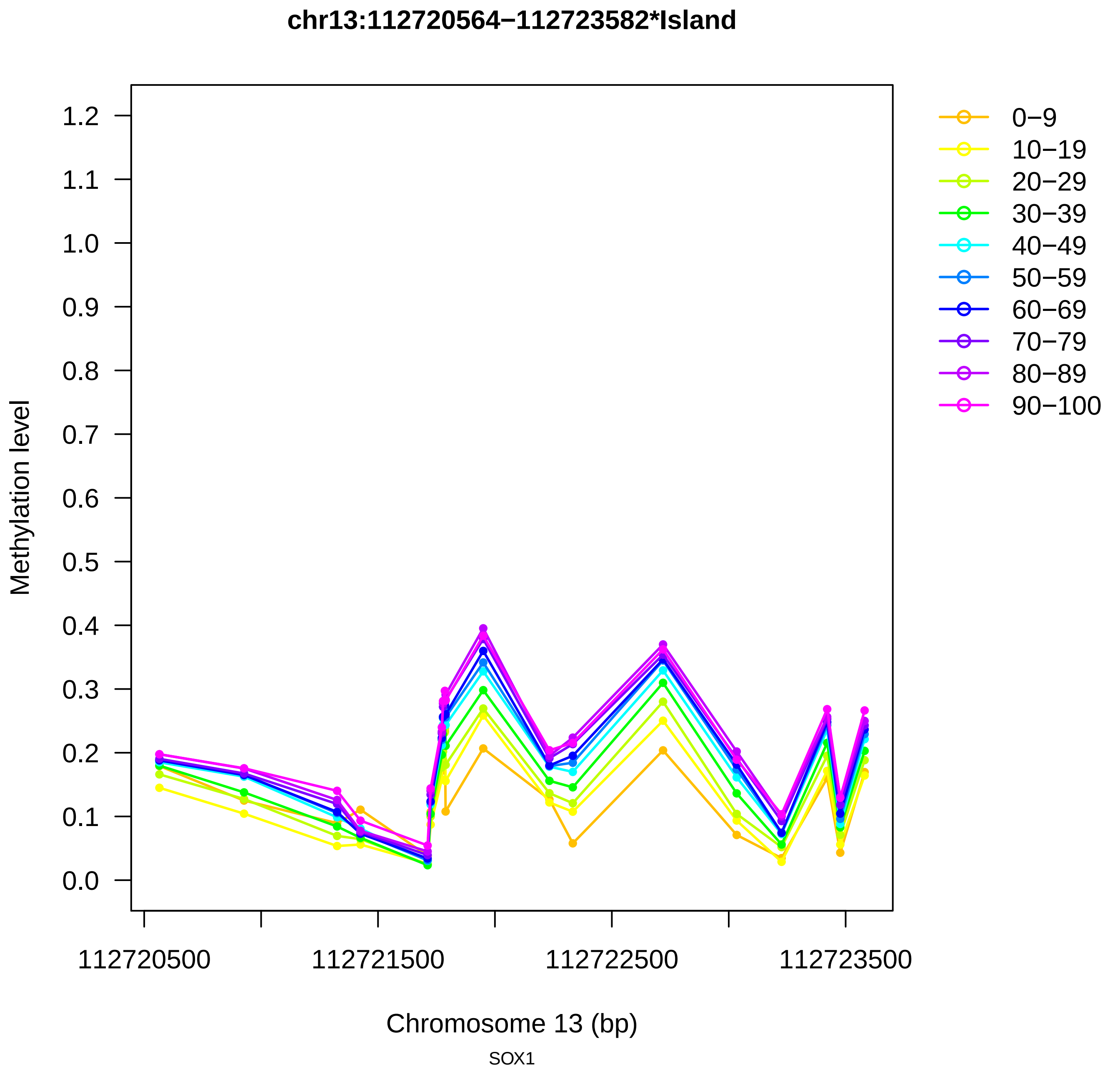

chr13:112720564−112723582*Island
Methylation level
0.0
0.1
0.2
0.3
0.4
0.5
0.6
0.7
0.8
0.9
1.0
1.1
1.2
112720500
112721500
112722500
112723500
Chromosome 13 (bp)
SOX1
0−9
10−19
20−29
30−39
40−49
50−59
60−69
70−79
80−89
90−100

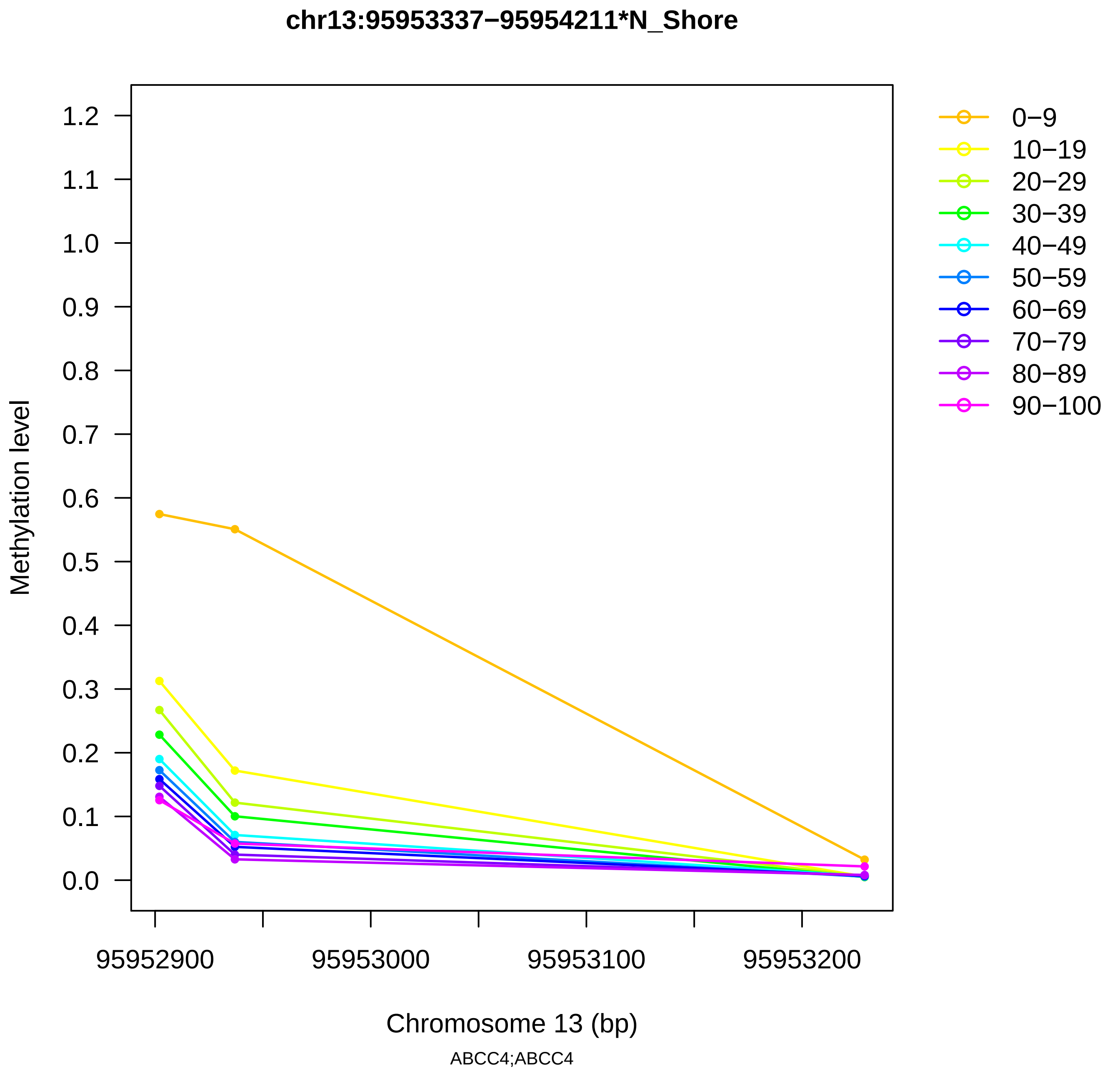
chr13:95953337−95954211*N_Shore
Methylation level
0.0
0.1
0.2
0.3
0.4
0.5
0.6
0.7
0.8
0.9
1.0
1.1
1.2
95952900
95953000
95953100
95953200
Chromosome 13 (bp)
ABCC4;ABCC4
0−9
10−19
20−29
30−39
40−49
50−59
60−69
70−79
80−89
90−100

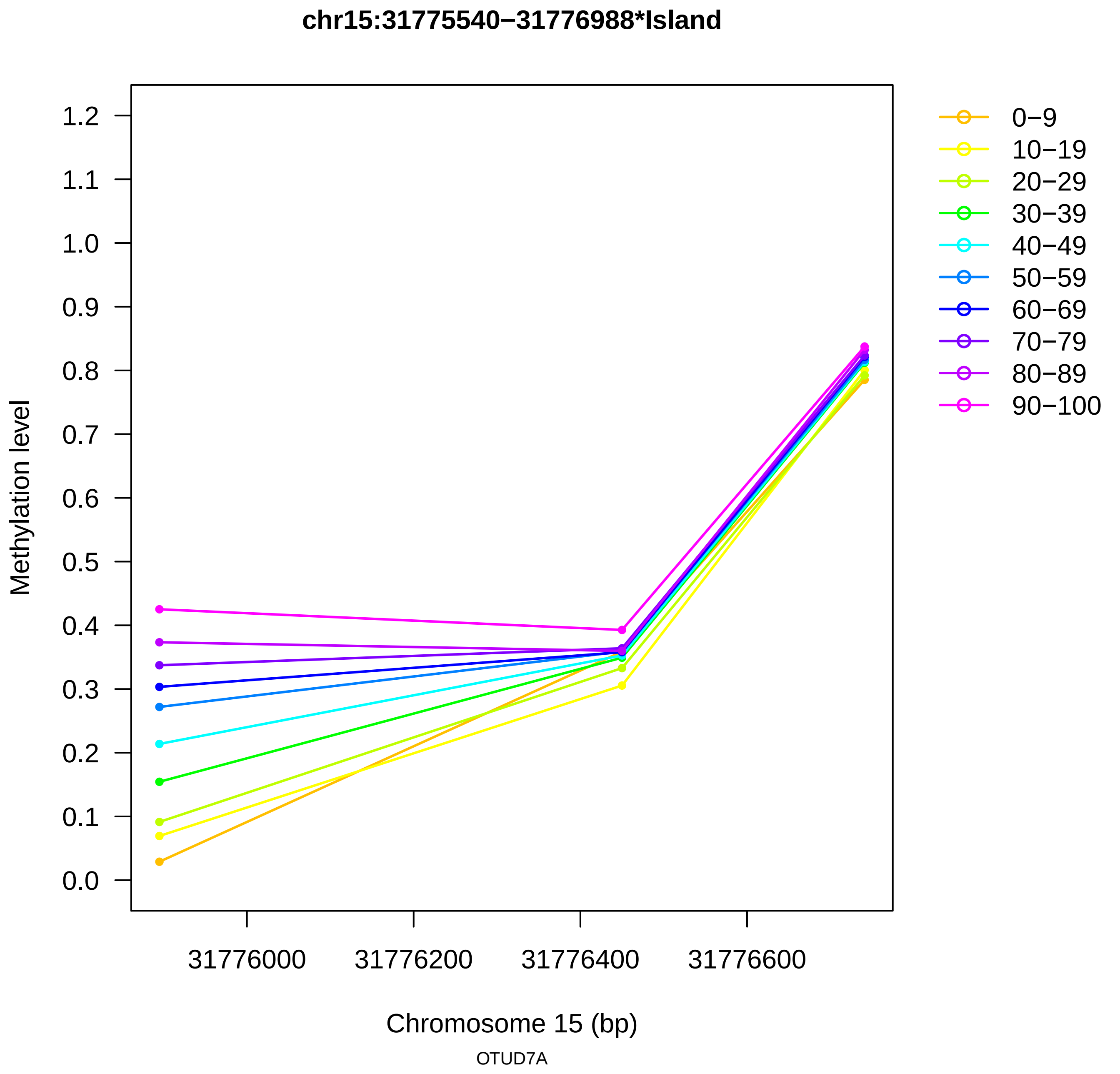

chr15:31775540−31776988*Island
Methylation level
1.2
1.1
1.0
0.9
0.8
0.7
0.6
0.5
0.4
0.3
0.2
0.1
0.0
31776000
31776200
31776400
31776600
Chromosome 15 (bp)
0−9
10−19
20−29
30−39
40−49
50−59
60−69
70−79
80−89
90−100
OTUD7A

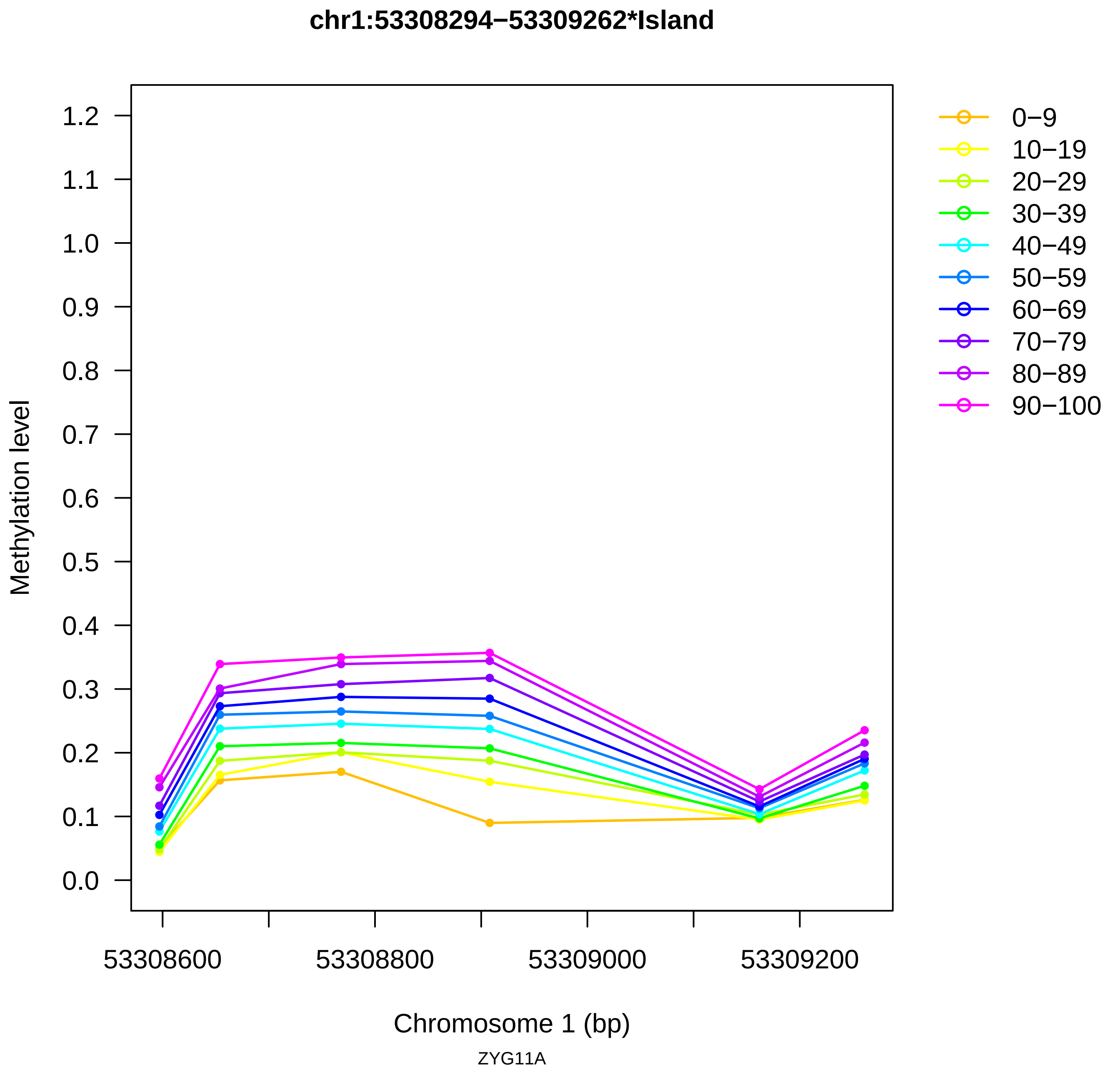
chr1:53308294−53309262*Island
Methylation level
1.2
1.1
1.0
0.9
0.8
0.7
0.6
0.5
0.4
0.3
0.2
0.1
0.0
53308600
53308800
53309000
53309200
Chromosome 1 (bp)
0−9
10−19
20−29
30−39
40−49
50−59
60−69
70−79
80−89
90−100
ZYG11A

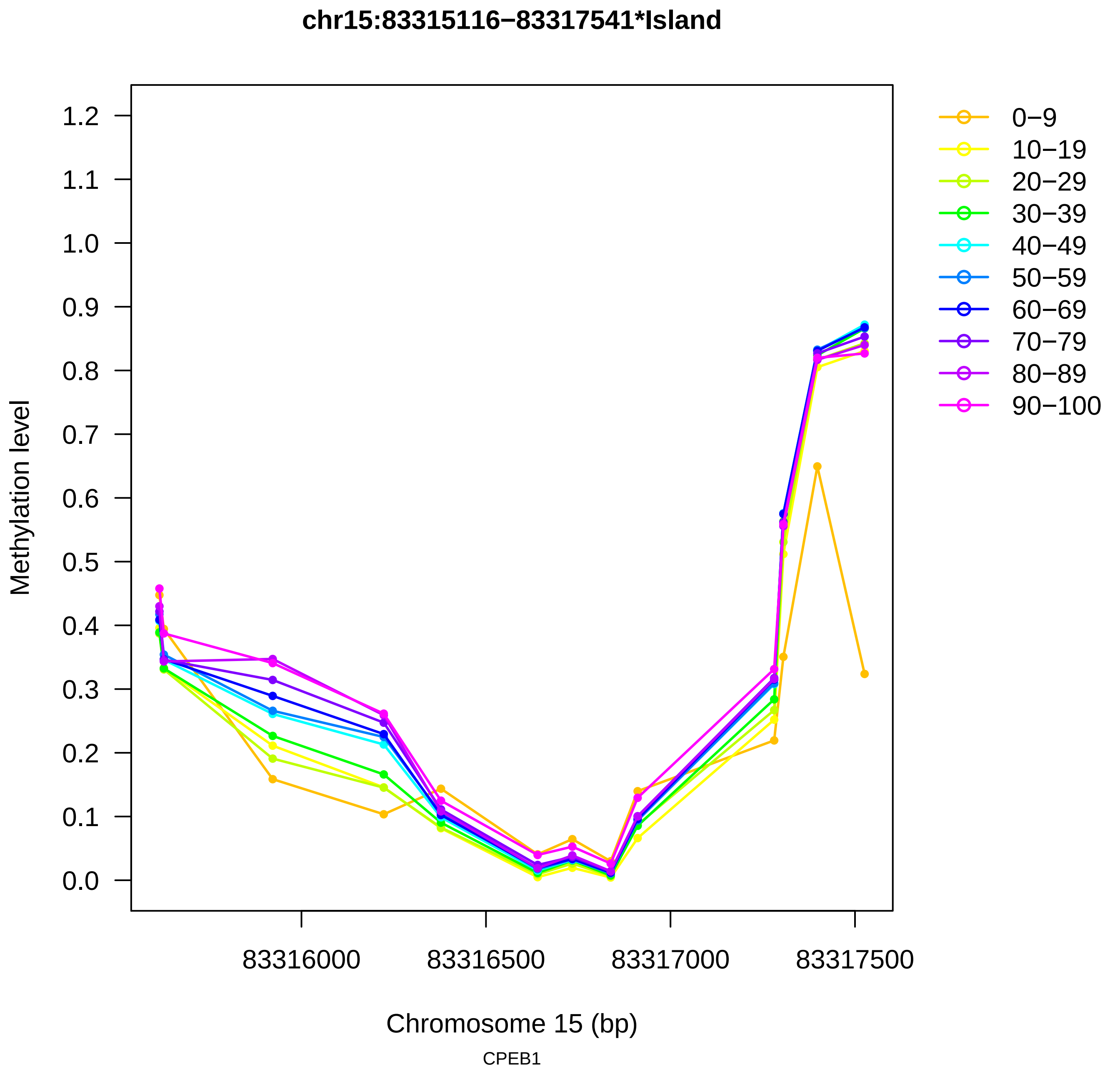

chr15:83315116−83317541*Island
Methylation level
0.0
0.1
0.2
0.3
0.4
0.5
0.6
0.7
0.8
0.9
1.0
1.1
1.2
83316000
83316500
83317000
83317500
Chromosome 15 (bp)
0−9
10−19
20−29
30−39
40−49
50−59
60−69
70−79
80−89
90−100

CPEB1

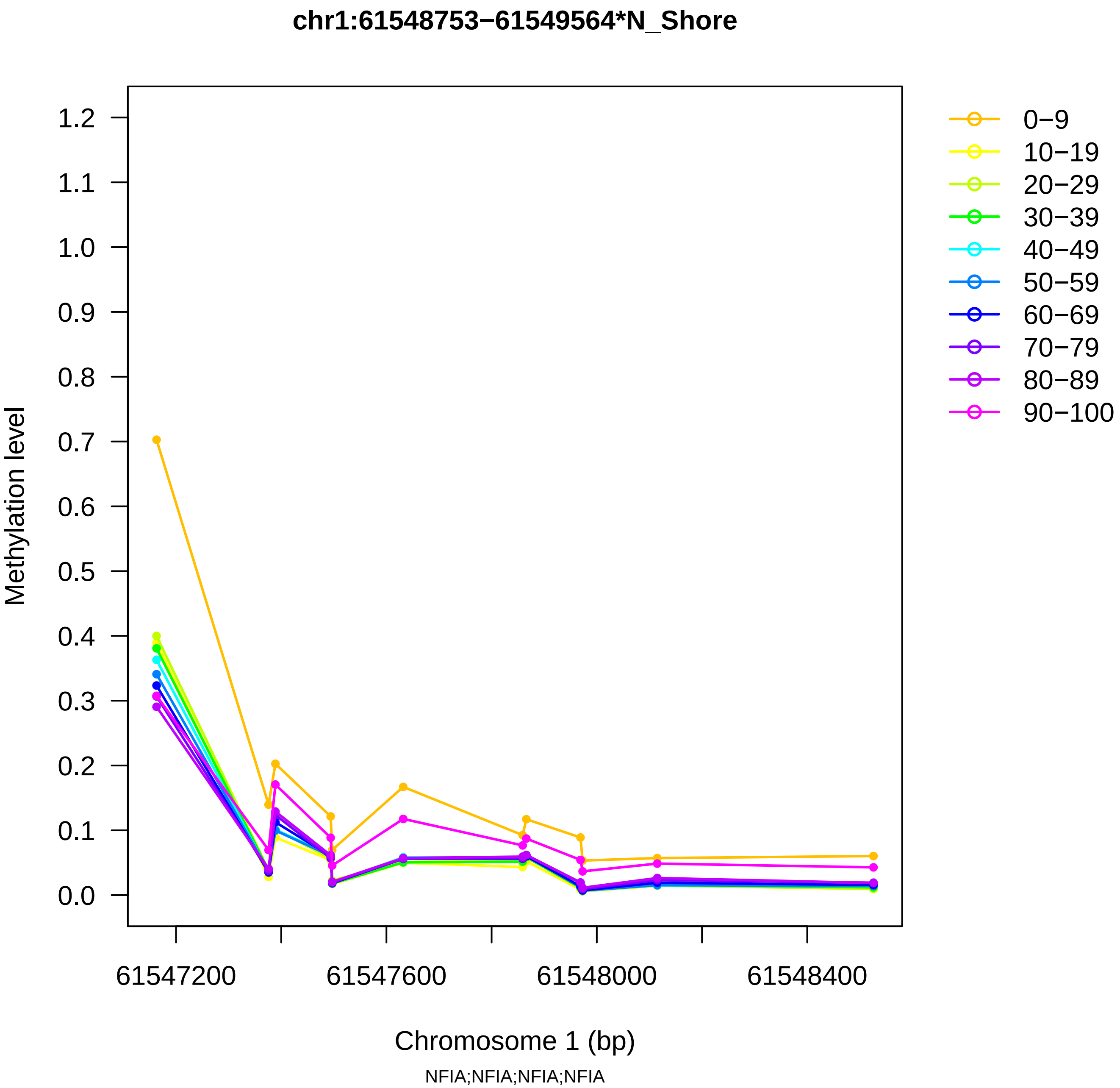
chr1:61548753−61549564*N_Shore
Methylation level
1.2
1.1
1.0
0.9
0.8
0.7
0.6
0.5
0.4
0.3
0.2
0.1
0.0
61547200
61547600
61548000
61548400
Chromosome 1 (bp)
NFIA;NFIA;NFIA;NFIA
0−9
10−19
20−29
30−39
40−49
50−59
60−69
70−79
80−89
90−100

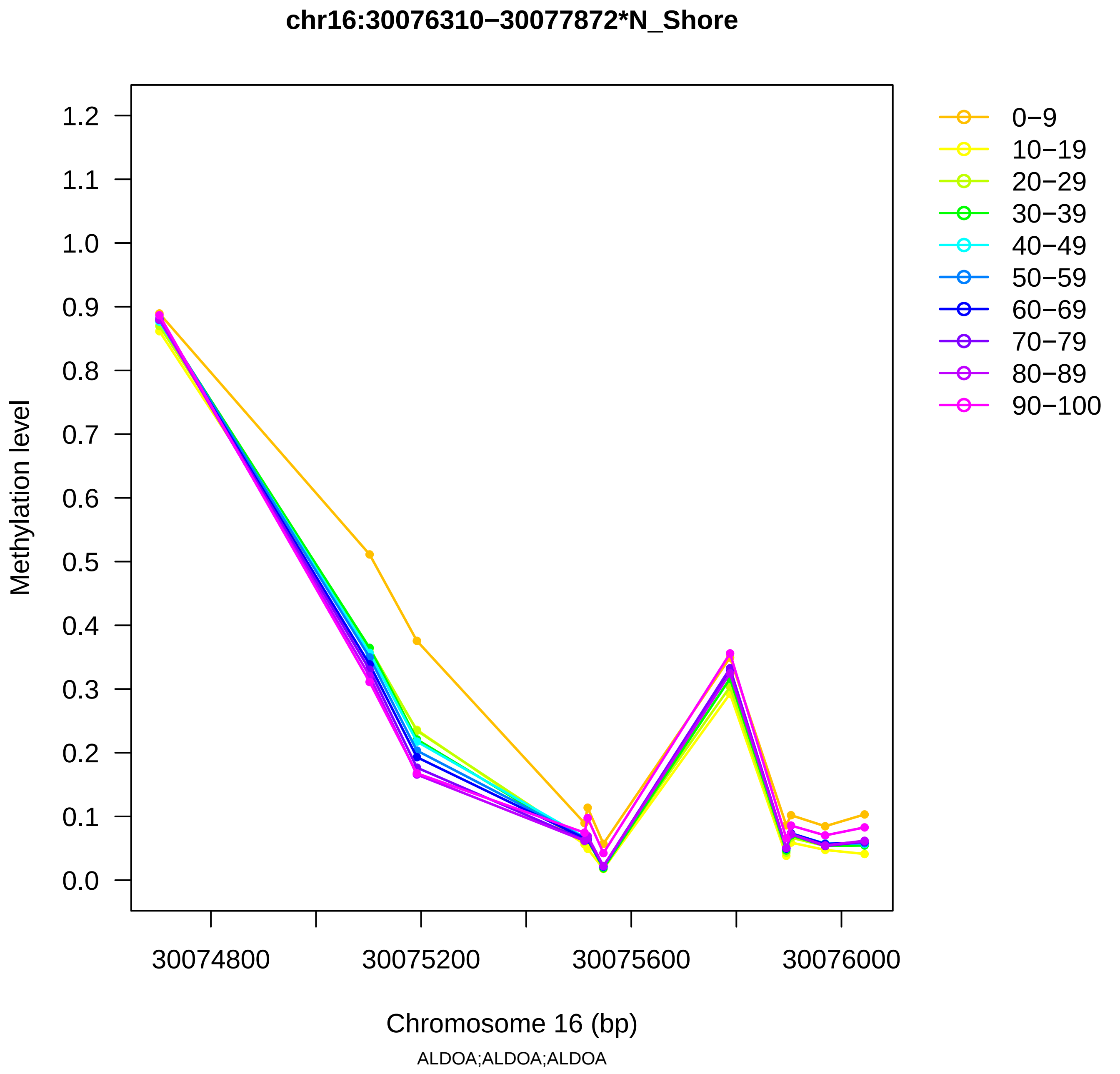
chr16:30076310−30077872*N_Shore
Methylation level
0.0
0.1
0.2
0.3
0.4
0.5
0.6
0.7
0.8
0.9
1.0
1.1
1.2
30074800
30075200
30075600
30076000
Chromosome 16 (bp)
ALDOA;ALDOA;ALDOA
0−9
10−19
20−29
30−39
40−49
50−59
60−69
70−79
80−89
90−100

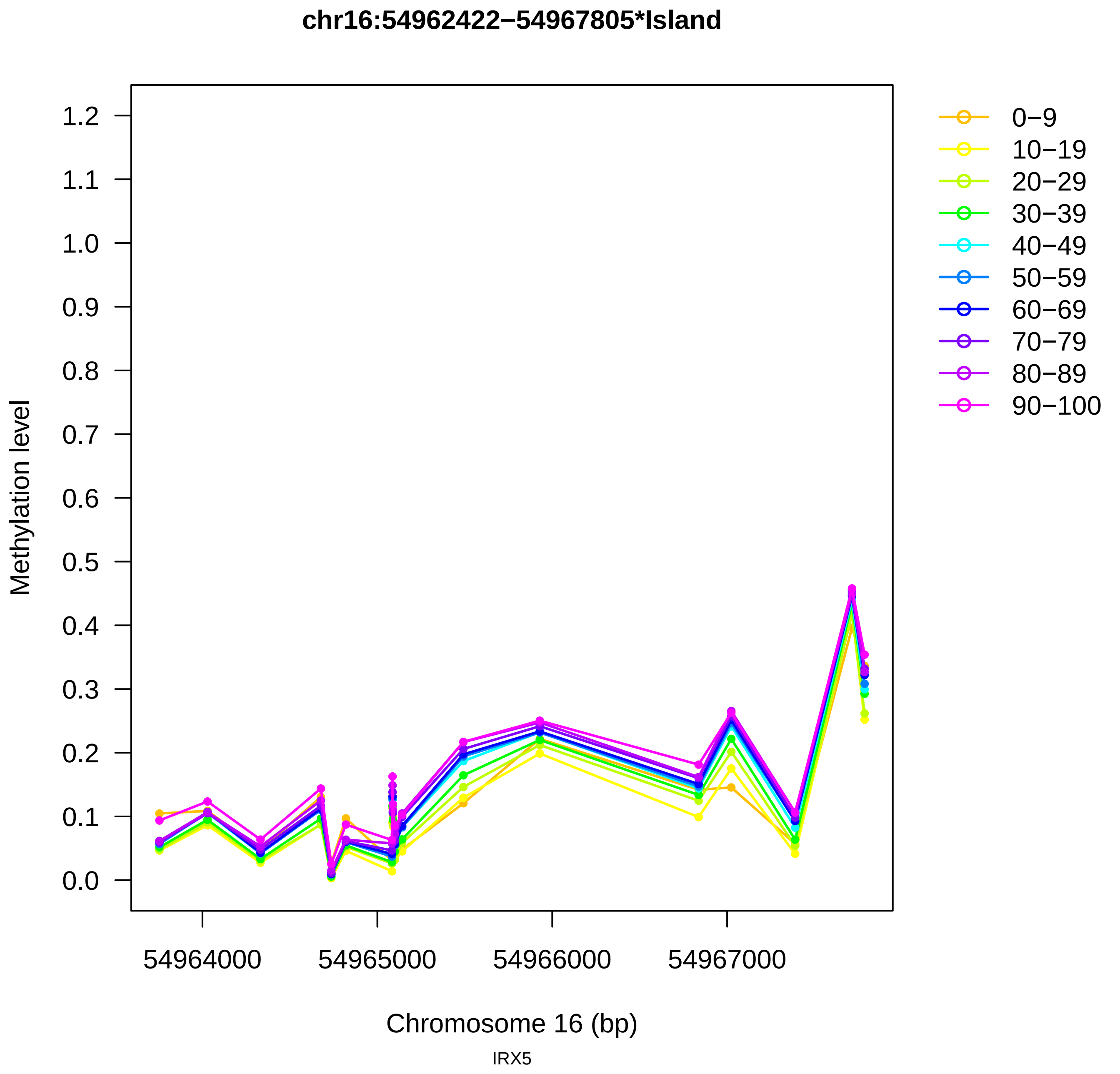
chr16:54962422−54967805*Island
Methylation level
0.0
0.1
0.2
0.3
0.4
0.5
0.6
0.7
0.8
0.9
1.0
1.1
1.2
54964000
54965000
54966000
54967000
Chromosome 16 (bp)
0−9
10−19
20−29
30−39
40−49
50−59
60−69
70−79
80−89
90−100
IRX5

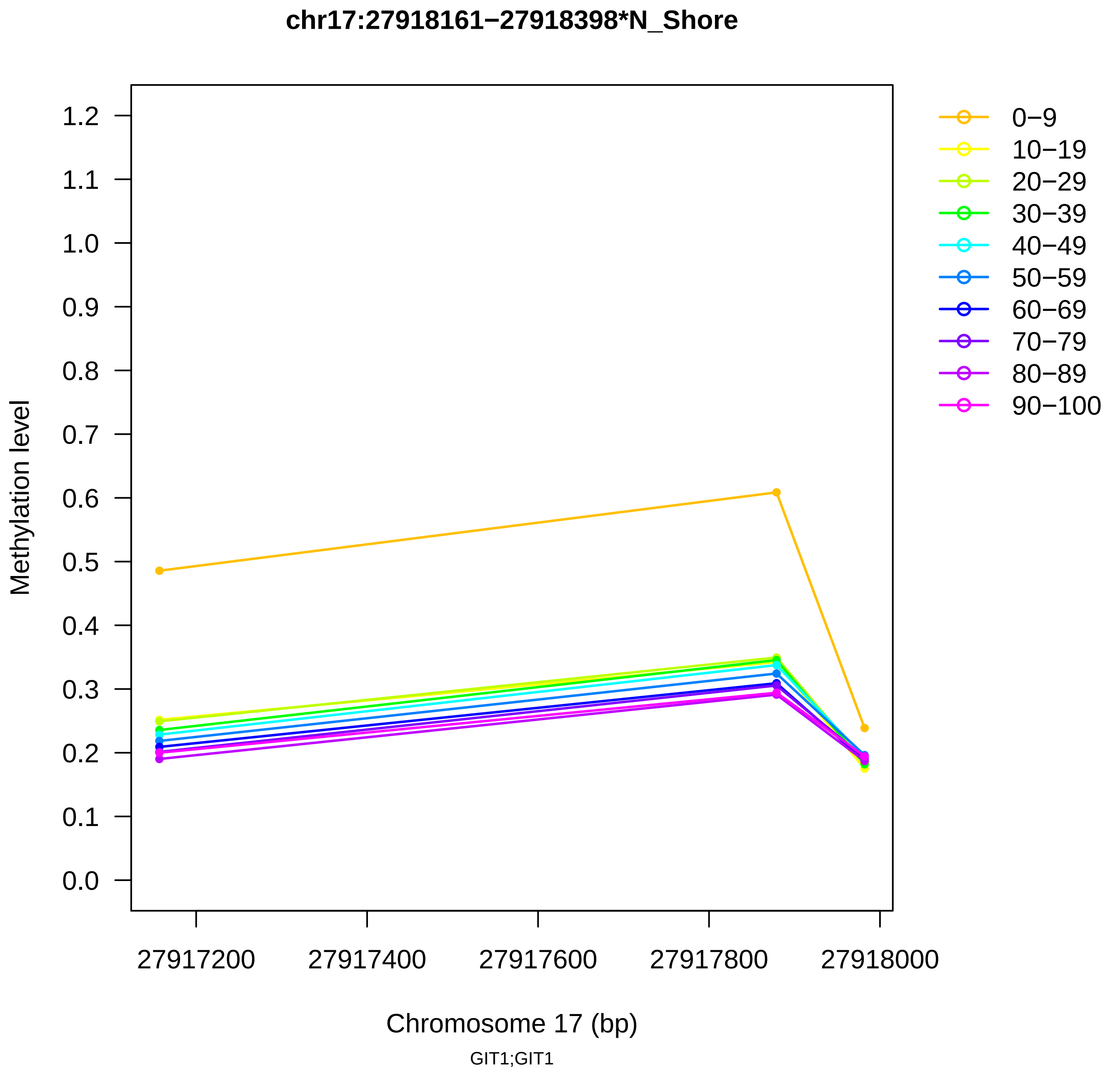
chr17:27918161−27918398*N_Shore
Methylation level
1.2
1.1
1.0
0.9
0.8
0.7
0.6
0.5
0.4
0.3
0.2
0.1
0.0
27917200
27917400
27917600
27917800
27918000
Chromosome 17 (bp)
GIT1;GIT1
0−9
10−19
20−29
30−39
40−49
50−59
60−69
70−79
80−89
90−100

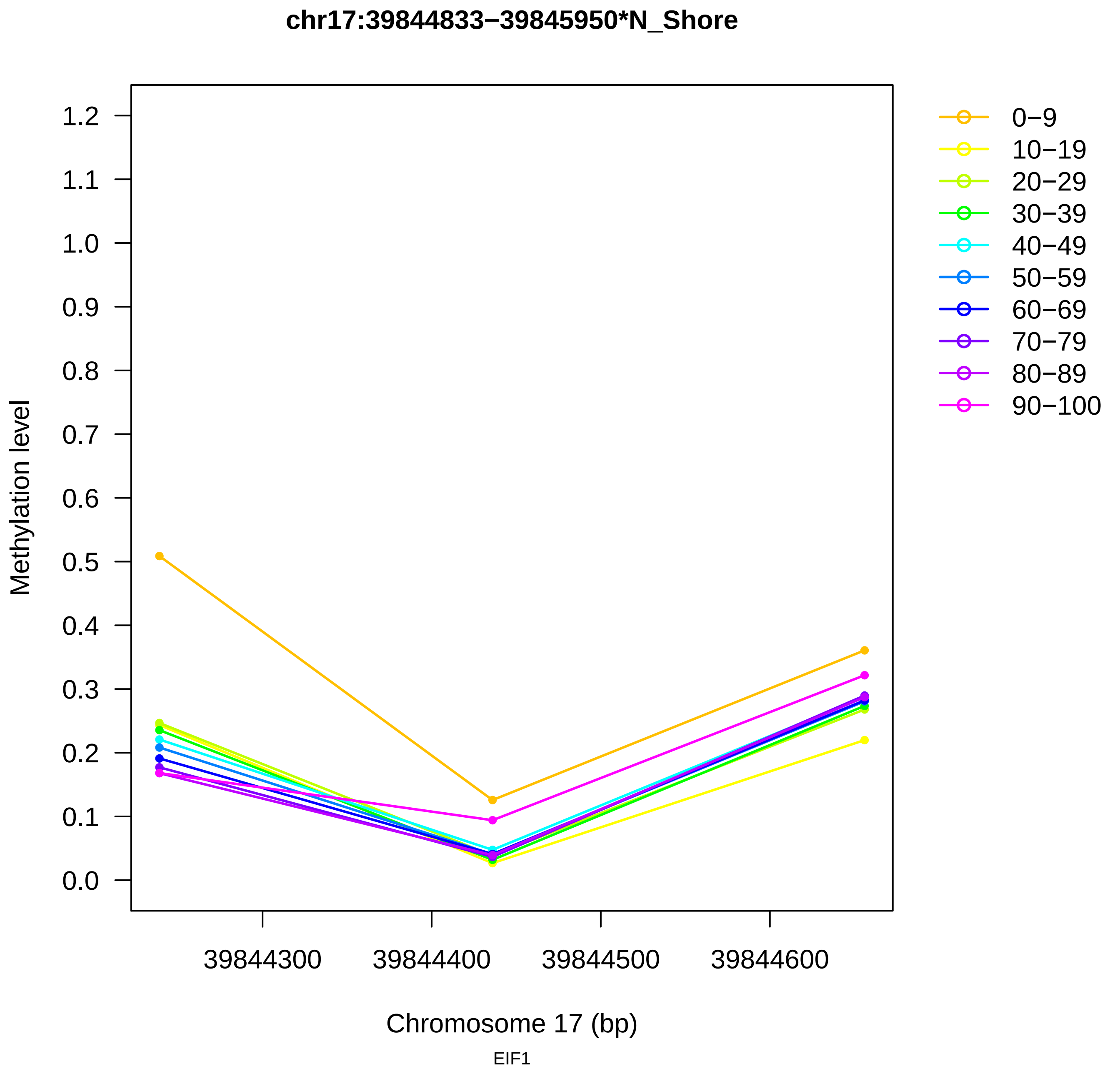
chr17:39844833−39845950*N_Shore
Methylation level
1.2
1.1
1.0
0.9
0.8
0.7
0.6
0.5
0.4
0.3
0.2
0.1
0.0
39844300
39844400
39844500
39844600
Chromosome 17 (bp)
EIF1
0−9
10−19
20−29
30−39
40−49
50−59
60−69
70−79
80−89
90−100

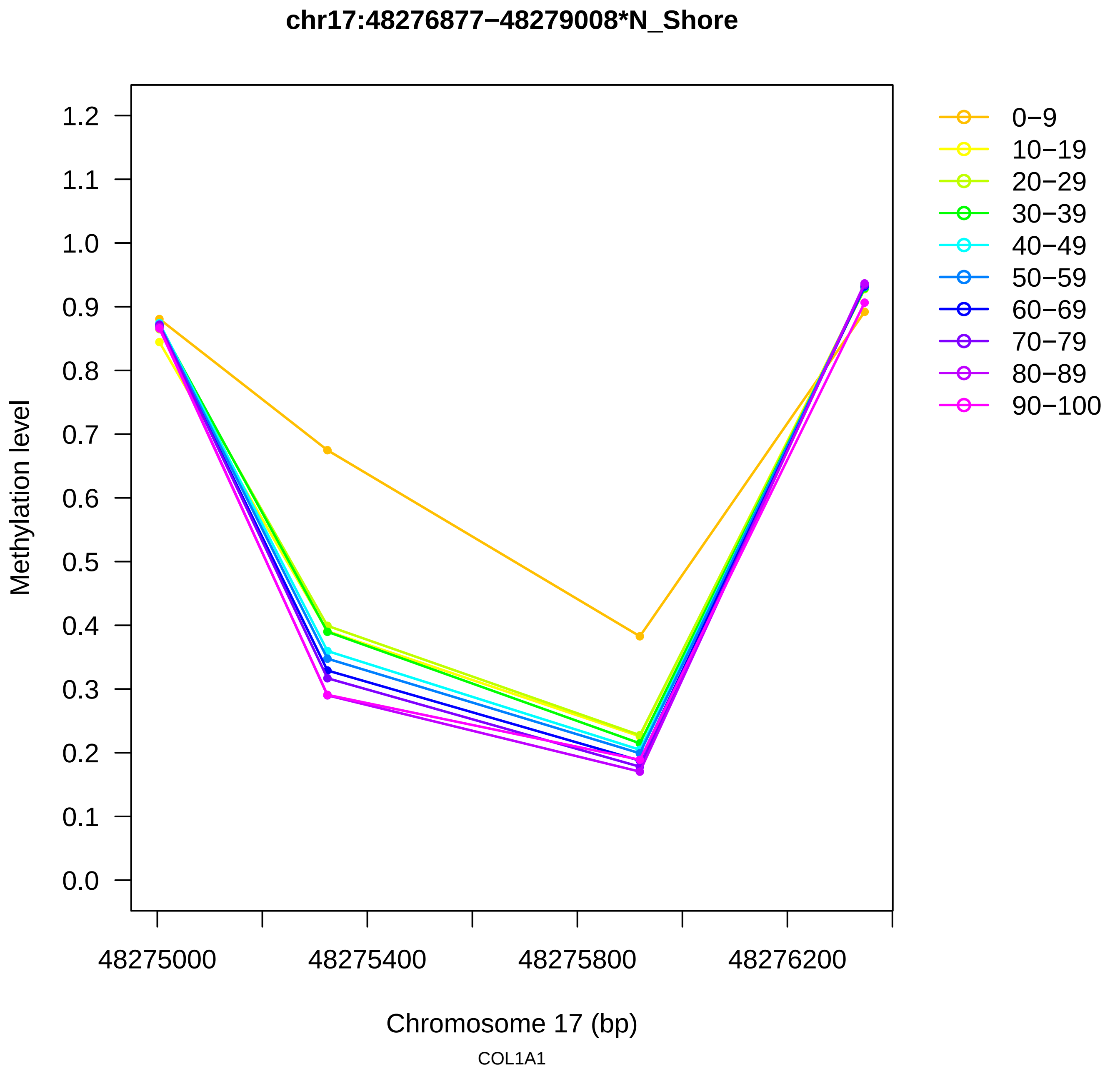
chr17:48276877−48279008*N_Shore
Methylation level
1.2
1.1
1.0
0.9
0.8
0.7
0.6
0.5
0.4
0.3
0.2
0.1
0.0
48275000
48275400
48275800
48276200
Chromosome 17 (bp)
COL1A1
0−9
10−19
20−29
30−39
40−49
50−59
60−69
70−79
80−89
90−100

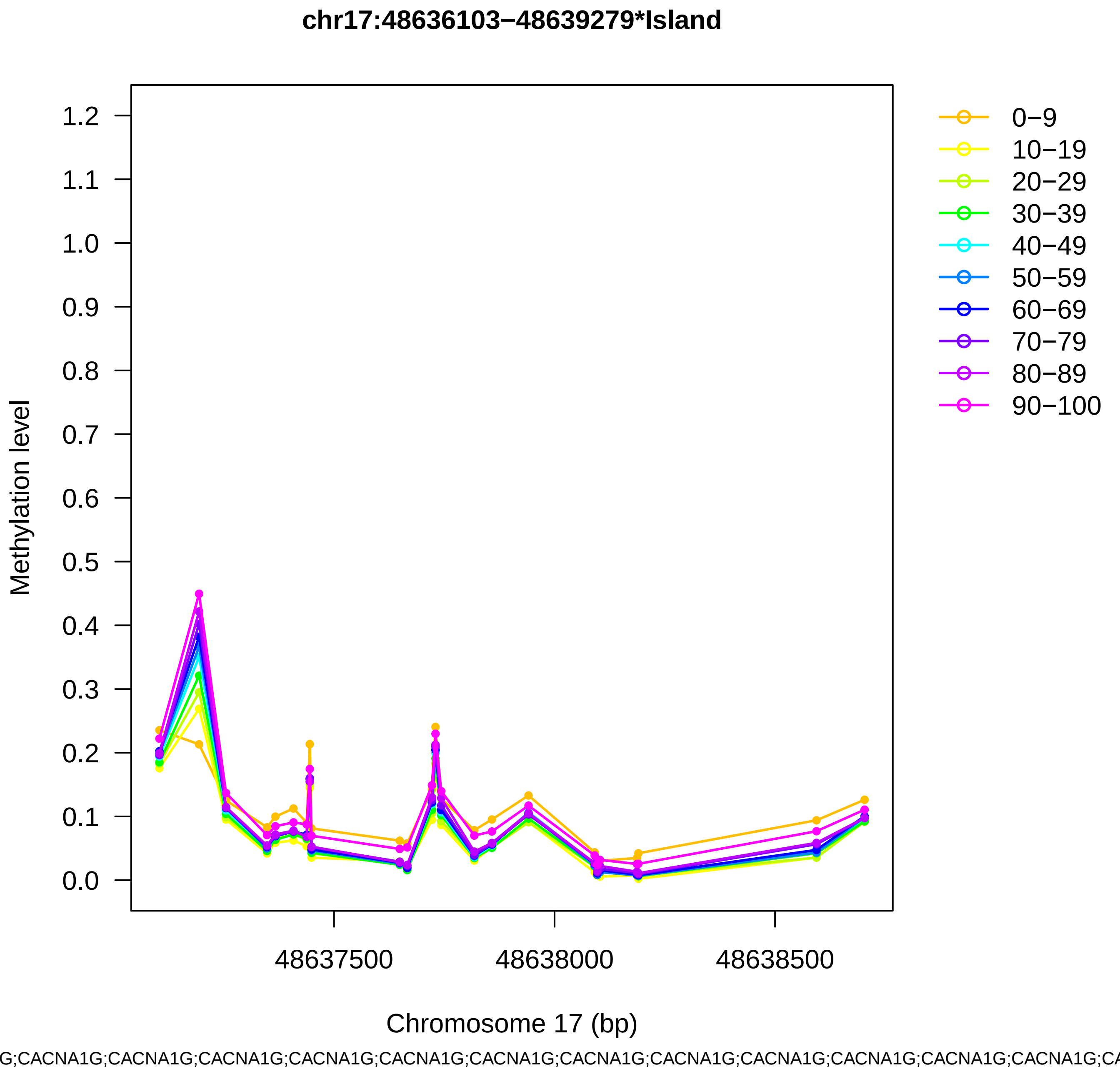
chr17:48636103−48639279*Island
Methylation level
1.2
1.1
1.0
0.9
0.8
0.7
0.6
0.5
0.4
0.3
0.2
0.1
0.0
48637500
48638000
48638500
Chromosome 17 (bp)
0−9
10−19
20−29
30−39
40−49
50−59
60−69
70−79
80−89
90−100
G;CACNA1G;CACNA1G;CACNA1G;CACNA1G;CACNA1G;CACNA1G;CACNA1G;CACNA1G;CACNA1G;CACNA1G;CACNA1G;CACNA1G;CACNA1G;CA

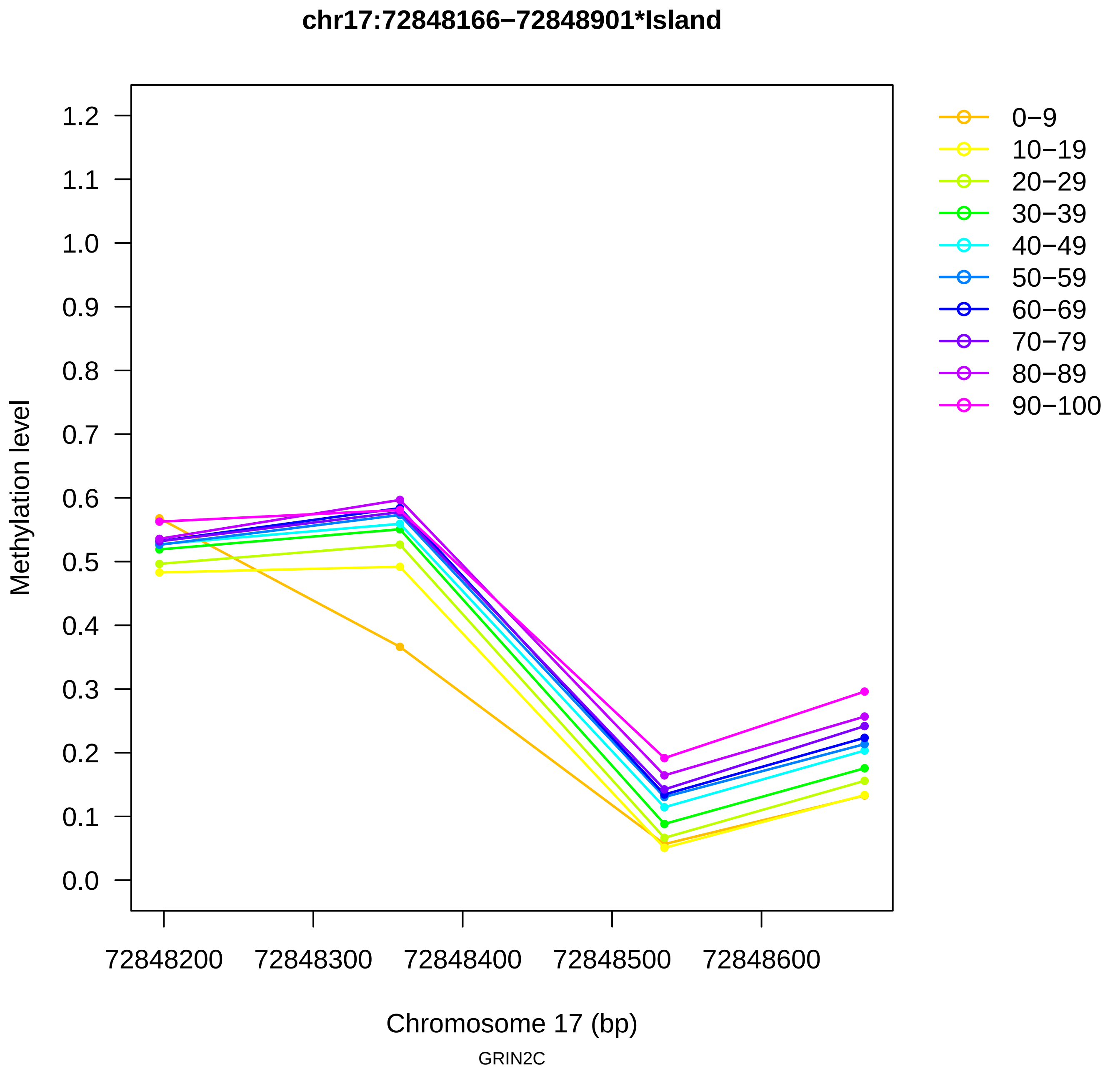
chr17:72848166−72848901*Island
Methylation level
0.0
0.1
0.2
0.3
0.4
0.5
0.6
0.7
0.8
0.9
1.0
1.1
1.2
72848200
72848300
72848400
72848500
72848600
Chromosome 17 (bp)
GRIN2C
0−9
10−19
20−29
30−39
40−49
50−59
60−69
70−79
80−89
90−100

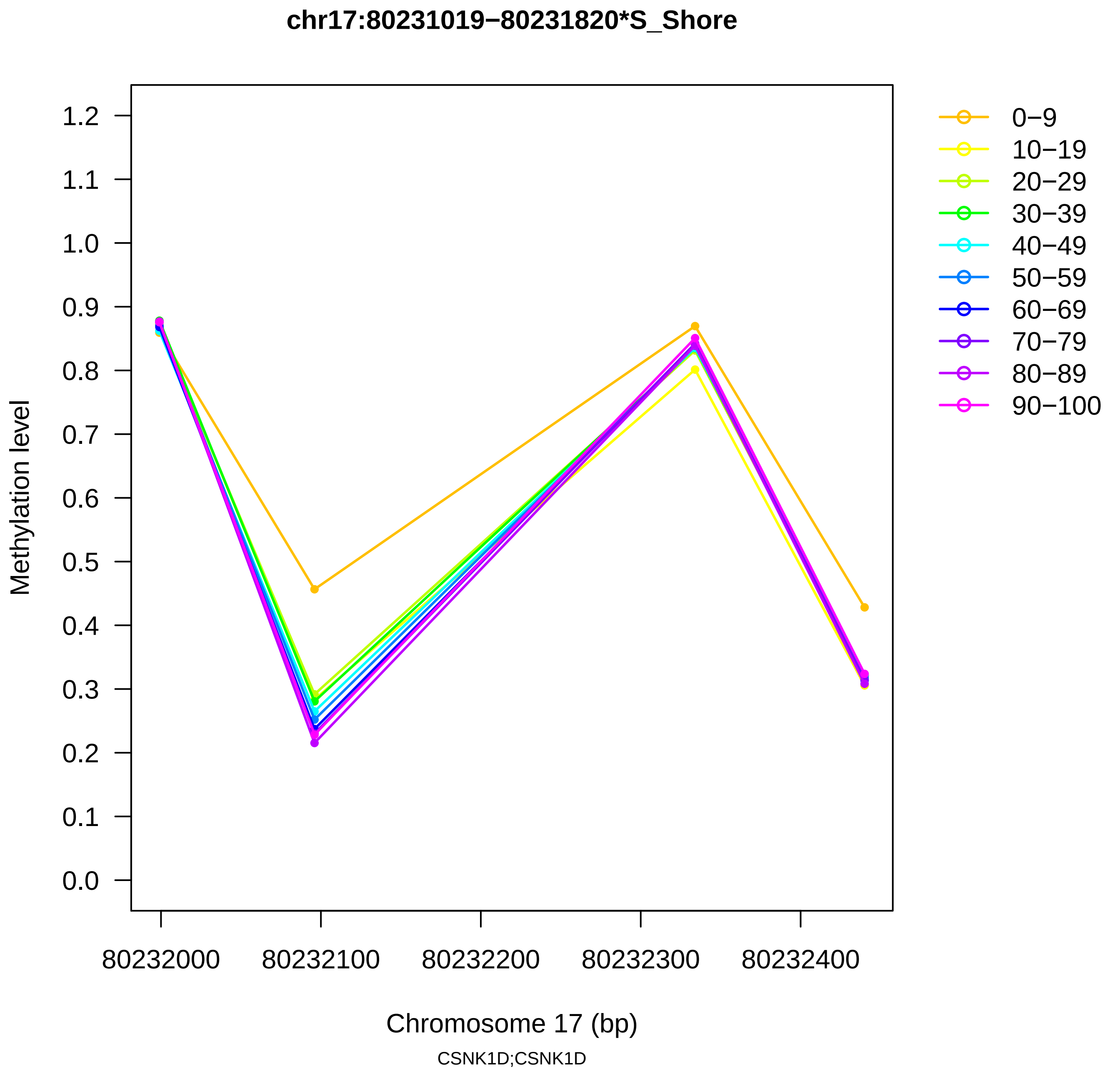
chr17:80231019−80231820*S_Shore
Methylation level
1.2
1.1
1.0
0.9
0.8
0.7
0.6
0.5
0.4
0.3
0.2
0.1
0.0
80232000
80232100
80232200
80232300
80232400
Chromosome 17 (bp)
CSNK1D;CSNK1D
0−9
10−19
20−29
30−39
40−49
50−59
60−69
70−79
80−89
90−100

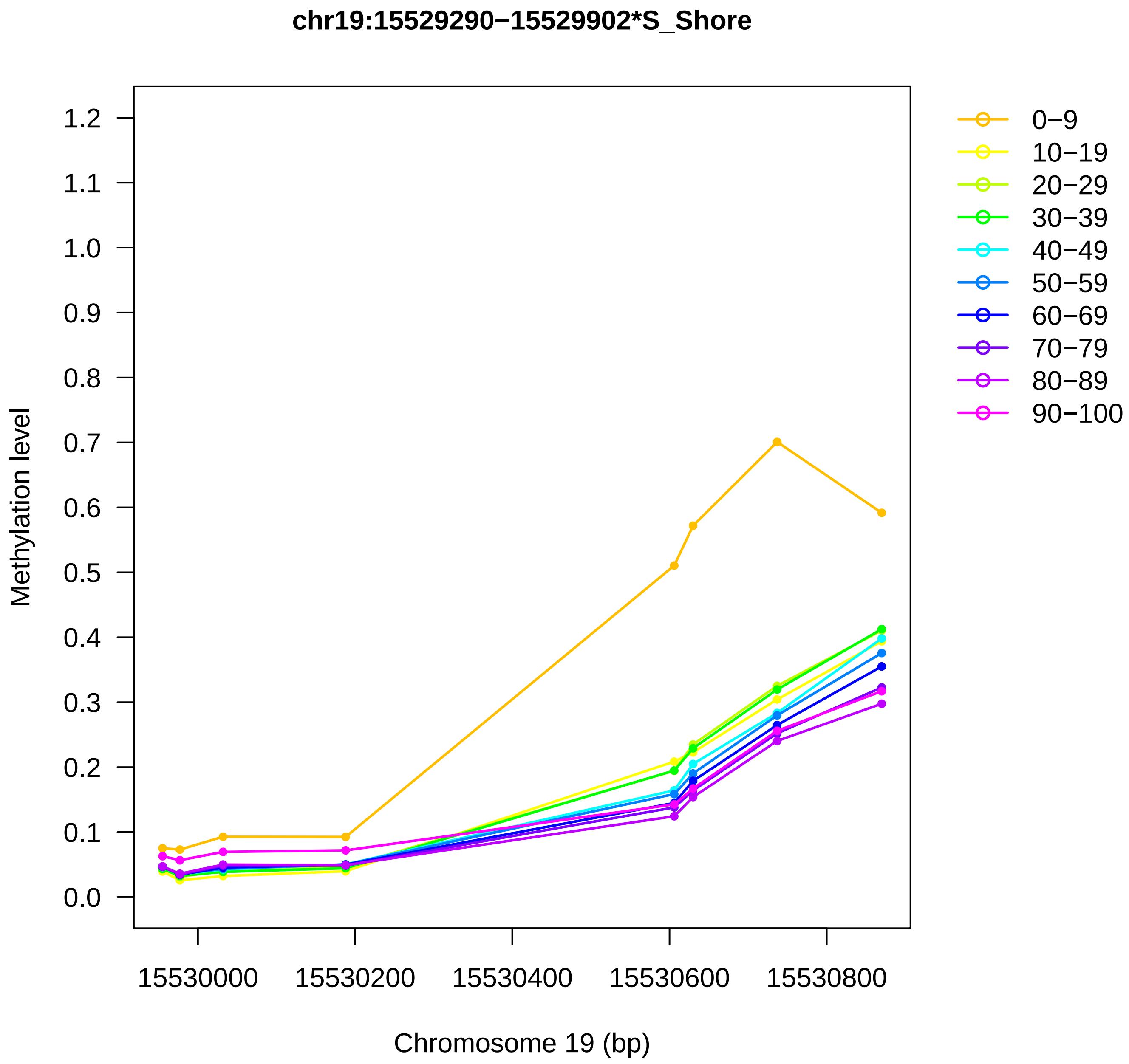

chr19:15529290−15529902*S_Shore
Methylation level
1.2
1.1
1.0
0.9
0.8
0.7
0.6
0.5
0.4
0.3
0.2
0.1
0.0
15530000
15530200
15530400
15530600
15530800
Chromosome 19 (bp)
0−9
10−19
20−29
30−39
40−49
50−59
60−69
70−79
80−89
90−100

AKAP8L

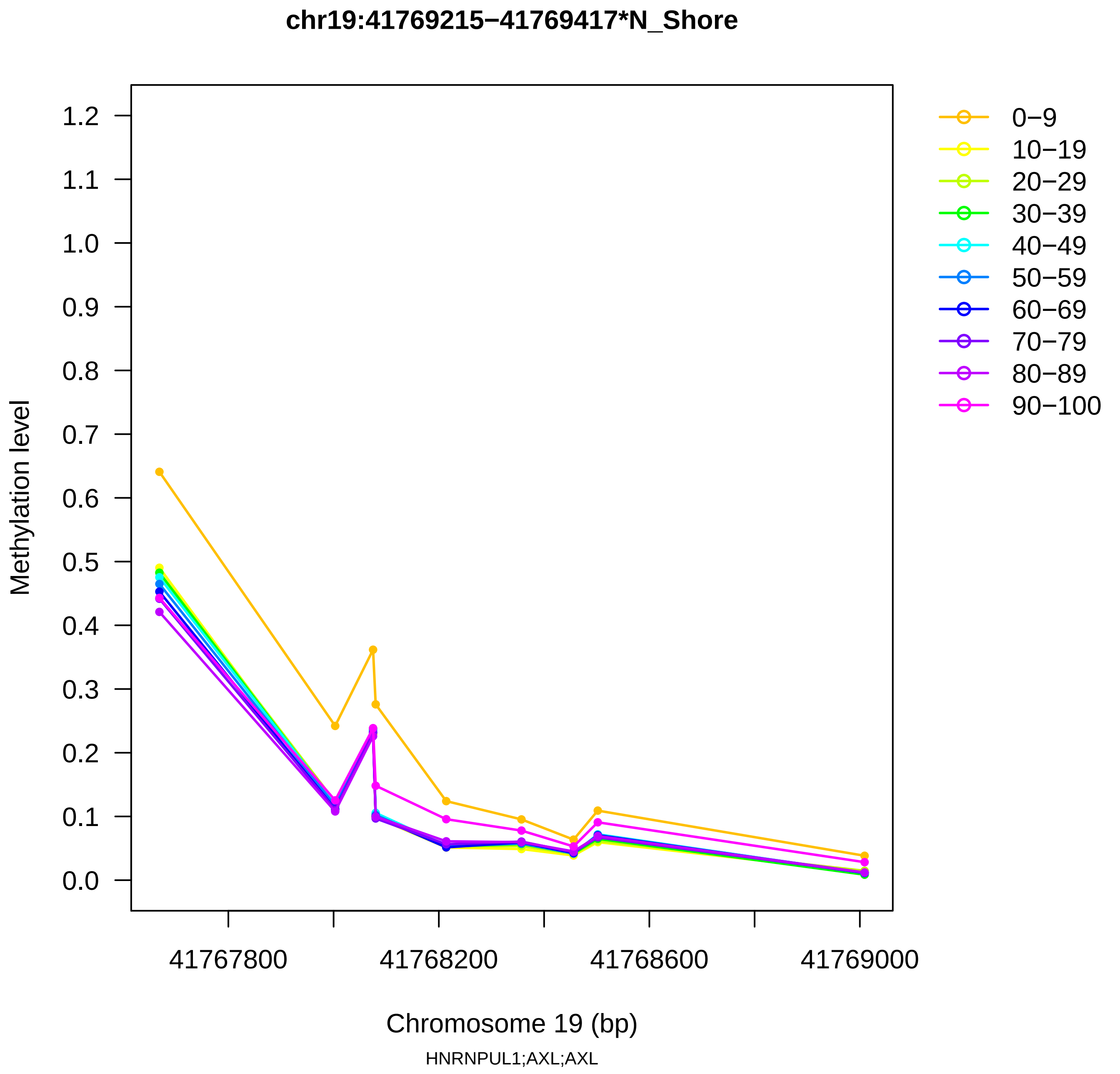

chr19:41769215−41769417*N_Shore
Methylation level
0.0
0.1
0.2
0.3
0.4
0.5
0.6
0.7
0.8
0.9
1.0
1.1
1.2
41767800
41768200
41768600
41769000
Chromosome 19 (bp)
HNRNPUL1;AXL;AXL
0−9
10−19
20−29
30−39
40−49
50−59
60−69
70−79
80−89
90−100

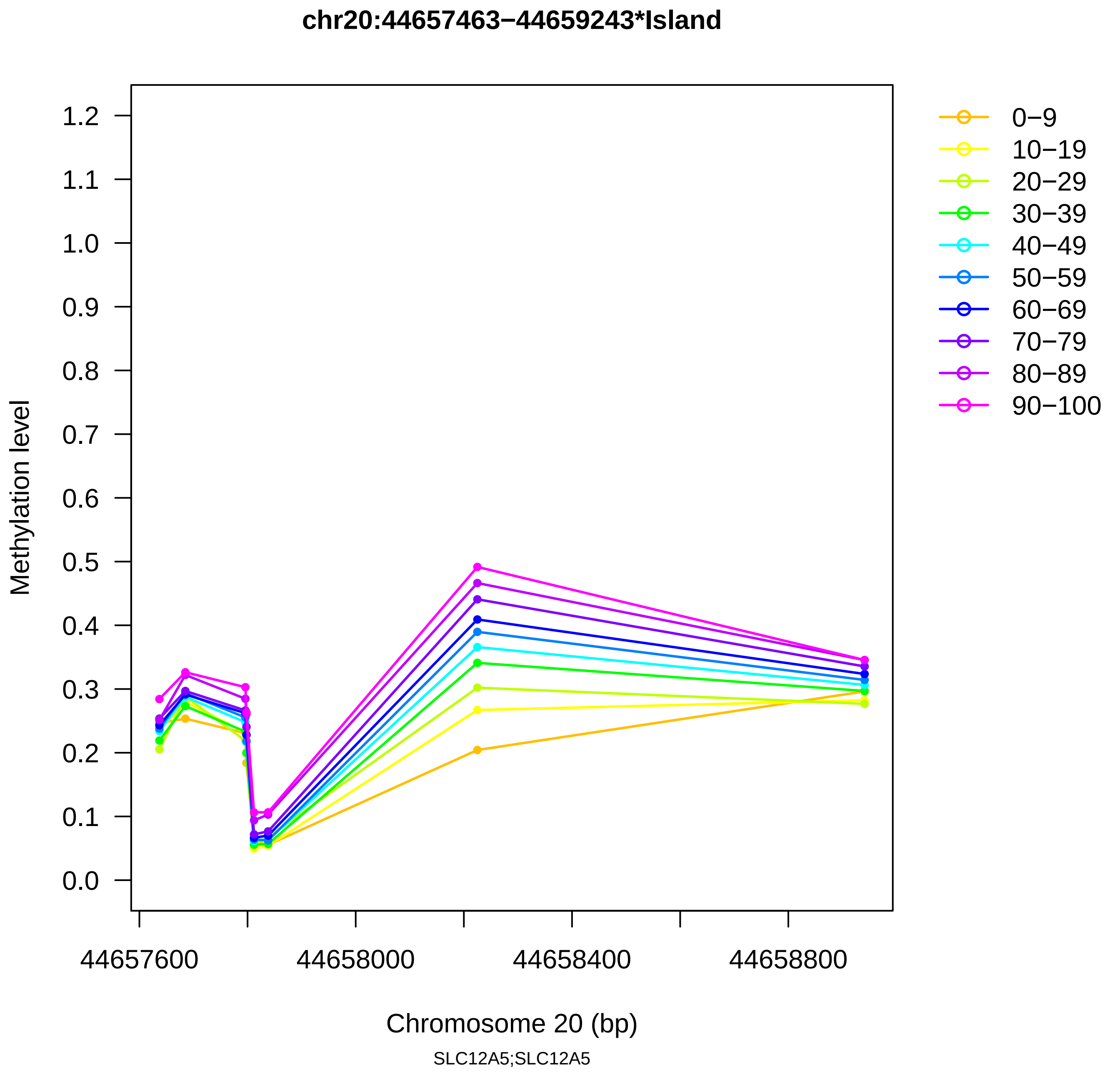

chr20:44657463−44659243*Island
Methylation level
0.0
0.1
0.2
0.3
0.4
0.5
0.6
0.7
0.8
0.9
1.0
1.1
1.2
44657600
44658000
44658400
44658800
Chromosome 20 (bp)
SLC12A5;SLC12A5
0−9
10−19
20−29
30−39
40−49
50−59
60−69
70−79
80−89
90−100

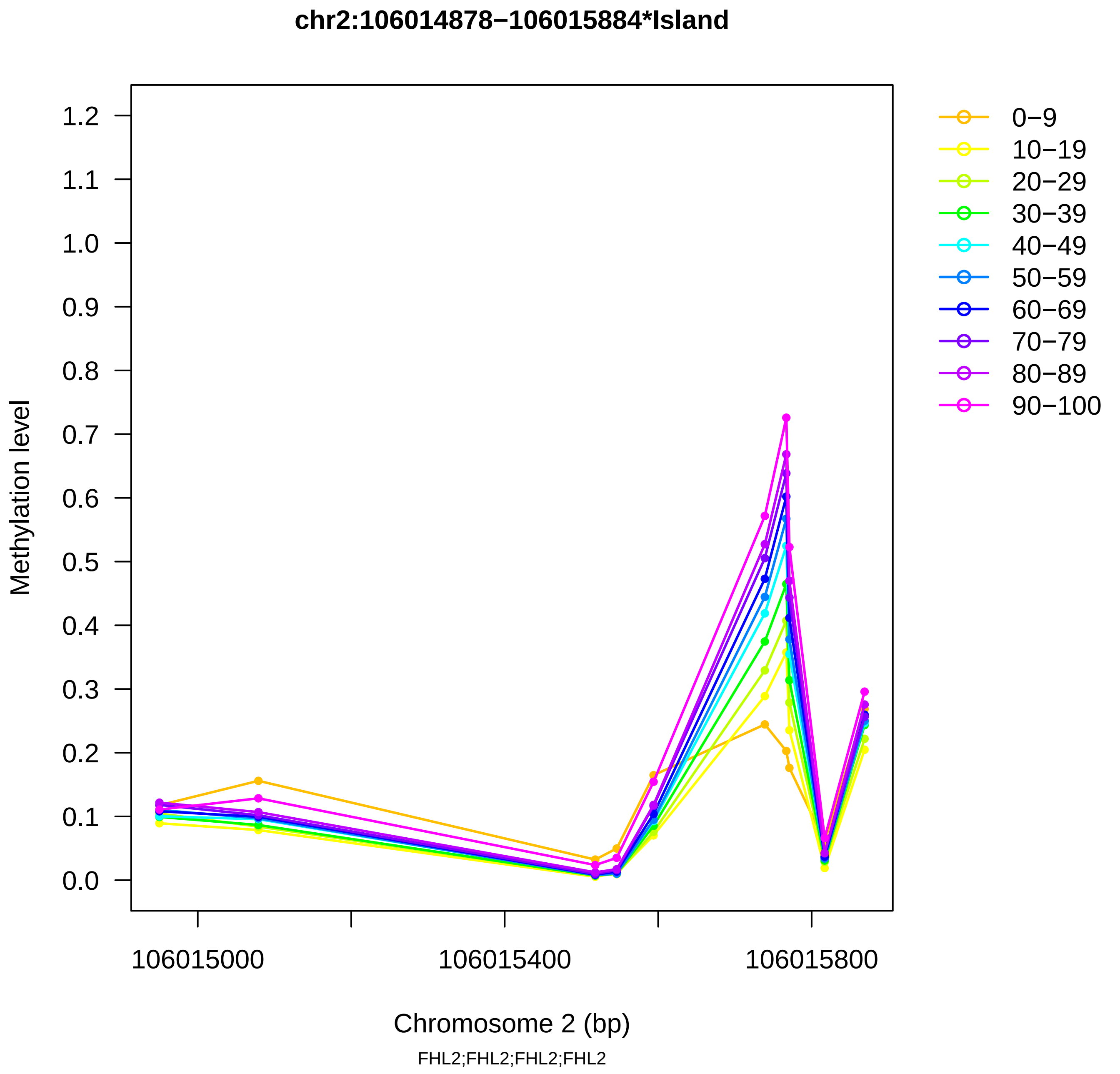
chr2:106014878−106015884*Island
Methylation level
0.0
0.1
0.2
0.3
0.4
0.5
0.6
0.7
0.8
0.9
1.0
1.1
1.2
106015000
106015400
106015800
Chromosome 2 (bp)
FHL2;FHL2;FHL2;FHL2
0−9
10−19
20−29
30−39
40−49
50−59
60−69
70−79
80−89
90−100

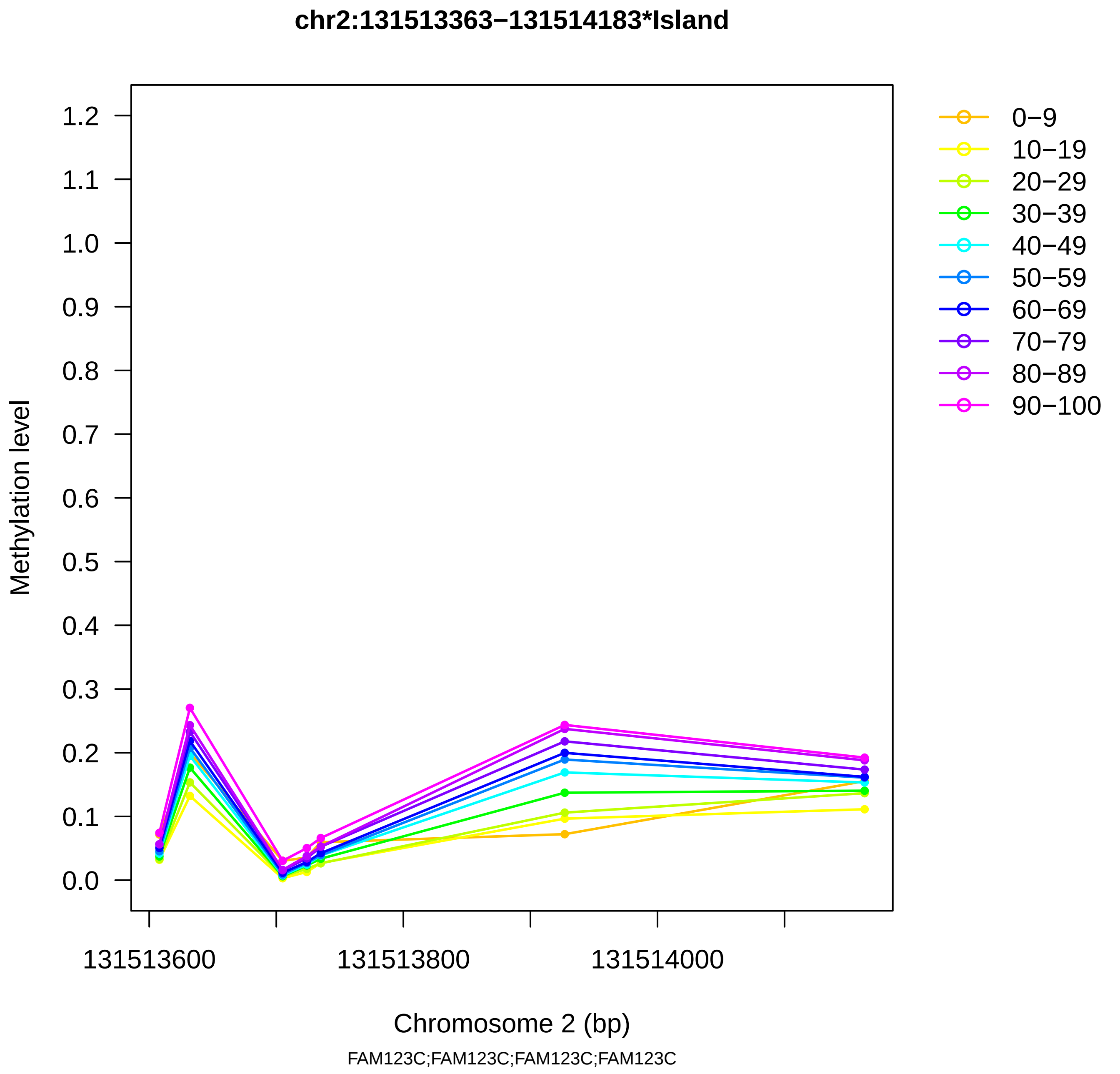
chr2:131513363−131514183*Island
Methylation level
0.0
0.1
0.2
0.3
0.4
0.5
0.6
0.7
0.8
0.9
1.0
1.1
1.2
131513600
131513800
131514000
Chromosome 2 (bp)
FAM123C;FAM123C;FAM123C;FAM123C
0−9
10−19
20−29
30−39
40−49
50−59
60−69
70−79
80−89
90−100

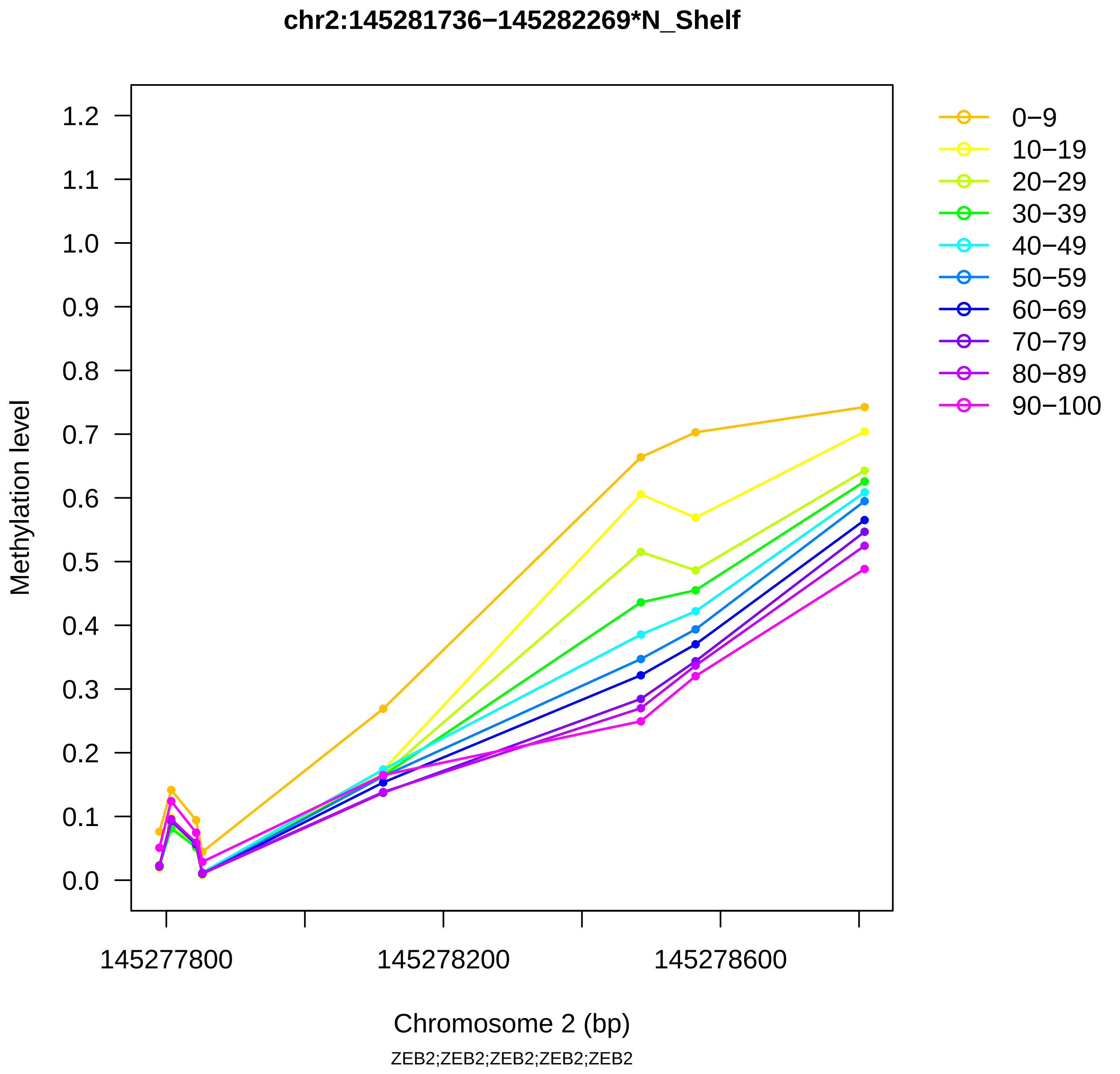
chr2:145281736−145282269*N_Shelf
Methylation level
0.0
0.1
0.2
0.3
0.4
0.5
0.6
0.7
0.8
0.9
1.0
1.1
1.2
145277800
145278200
145278600
Chromosome 2 (bp)
ZEB2;ZEB2;ZEB2;ZEB2;ZEB2
0−9
10−19
20−29
30−39
40−49
50−59
60−69
70−79
80−89
90−100

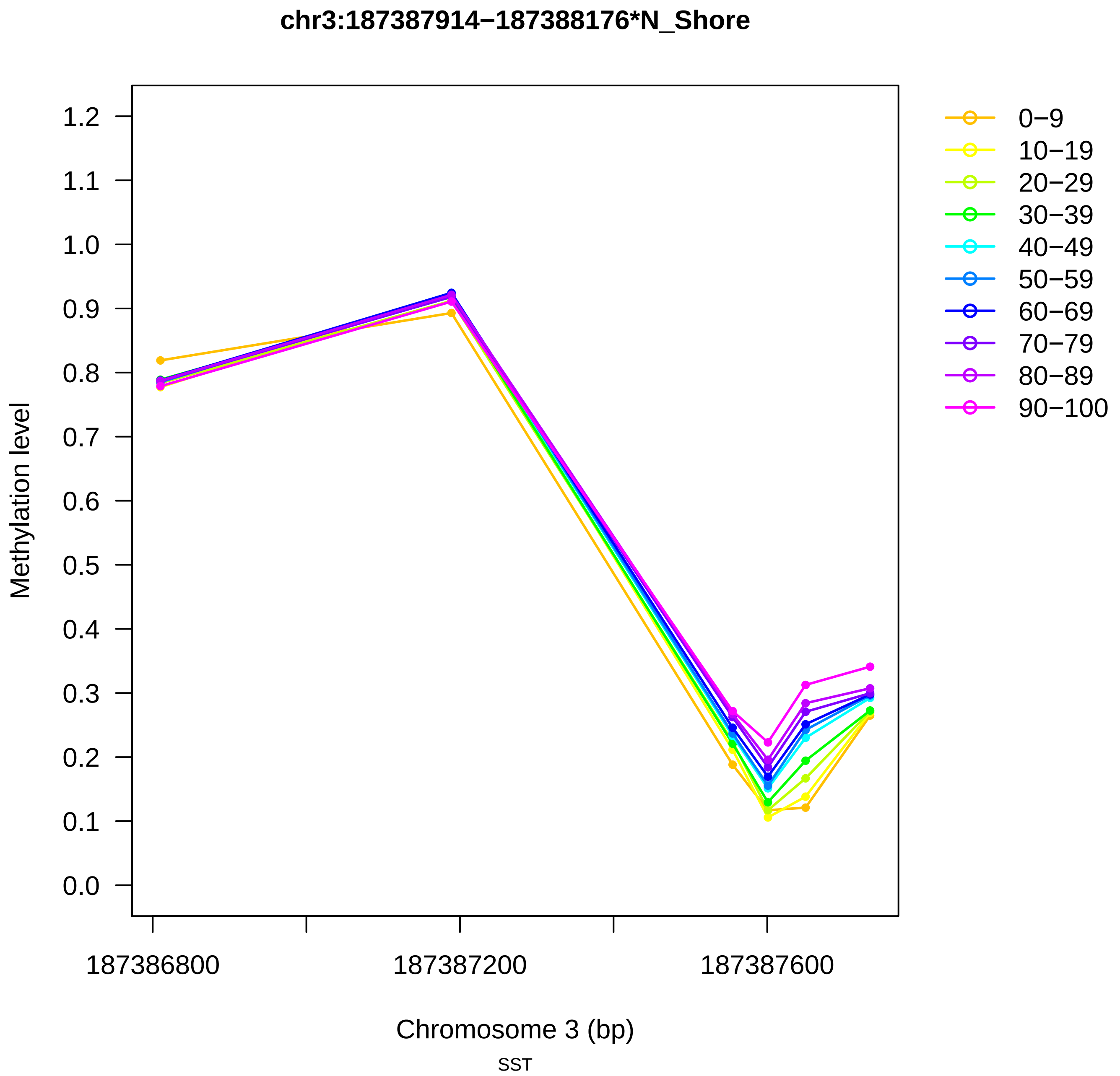
chr3:187387914−187388176*N_Shore
Methylation level
Chromosome 3 (bp)
SST
0−9
10−19
20−29
30−39
40−49
50−59
60−69
70−79
80−89
90−100
187386800
187387200
187387600
0.0
0.1
0.2
0.3
0.4
0.5
0.6
0.7
0.8
0.9
1.0
1.1
1.2

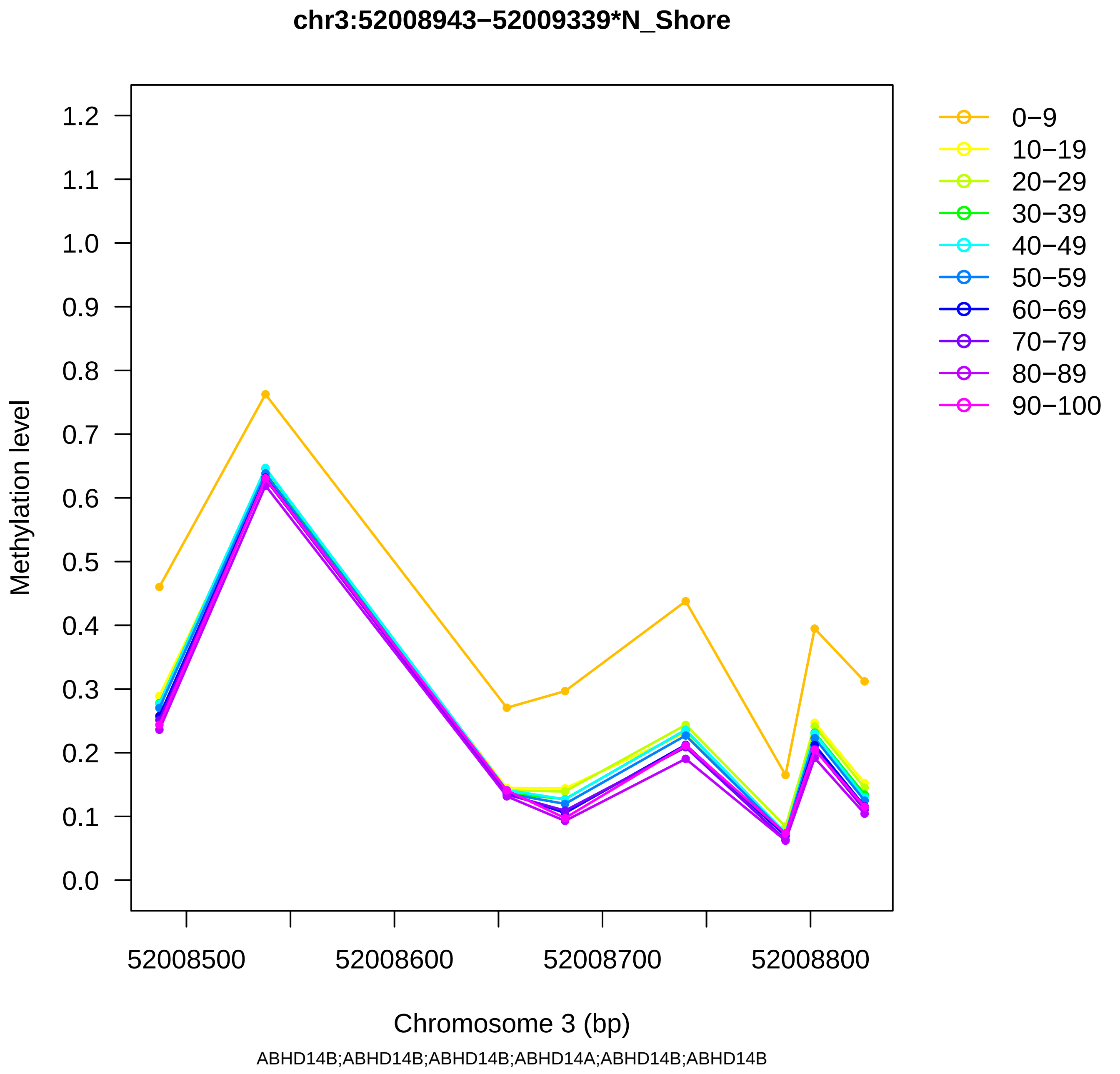
chr3:52008943−52009339*N_Shore
Methylation level
0.0
0.1
0.2
0.3
0.4
0.5
0.6
0.7
0.8
0.9
1.0
1.1
1.2
52008500
52008600
52008700
52008800
Chromosome 3 (bp)
ABHD14B;ABHD14B;ABHD14B;ABHD14A;ABHD14B;ABHD14B
0−9
10−19
20−29
30−39
40−49
50−59
60−69
70−79
80−89
90−100

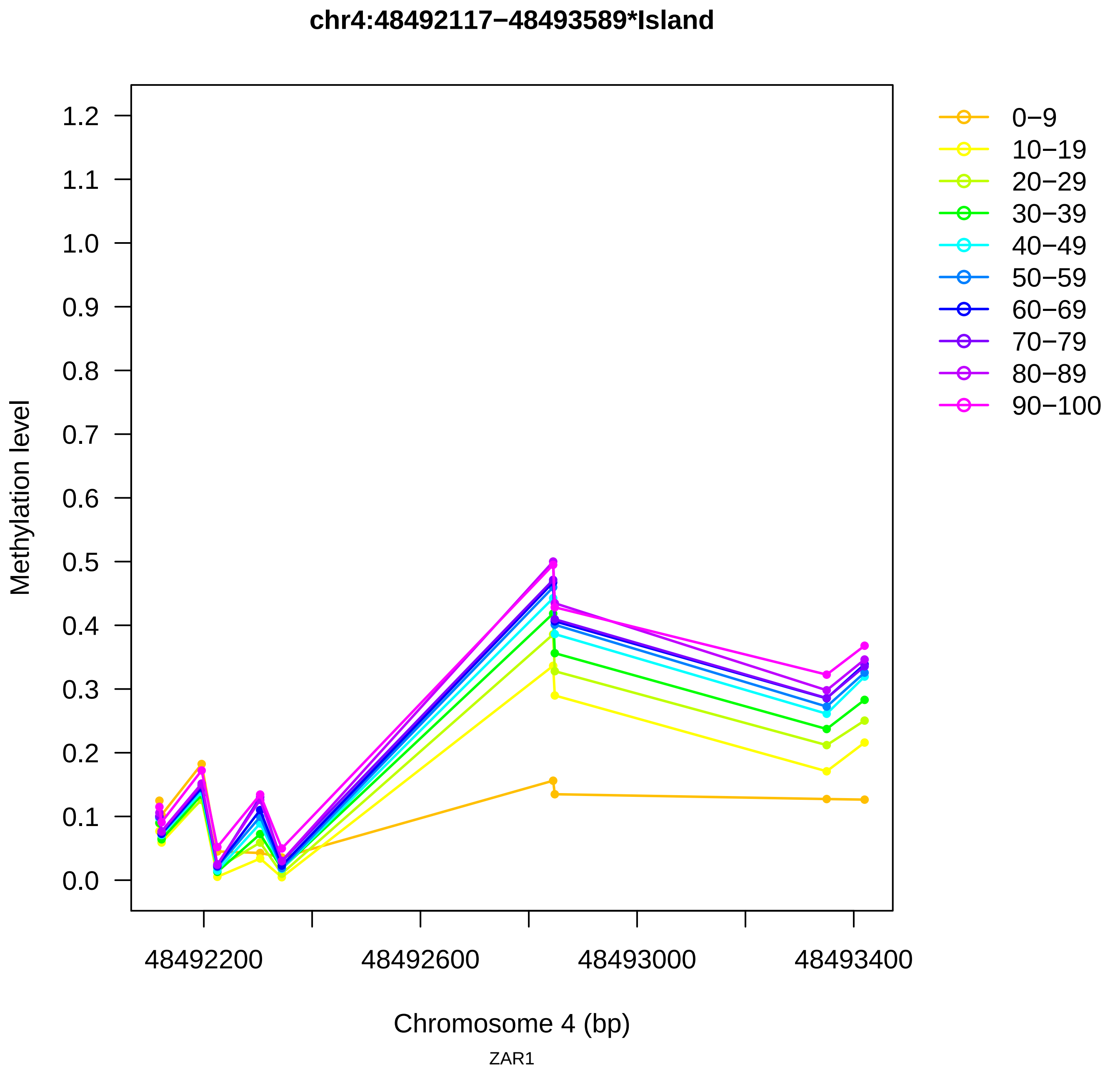
chr4:48492117−48493589*Island
Methylation level
1.2
1.1
1.0
0.9
0.8
0.7
0.6
0.5
0.4
0.3
0.2
0.1
0.0
48492200
48492600
48493000
48493400
Chromosome 4 (bp)
0−9
10−19
20−29
30−39
40−49
50−59
60−69
70−79
80−89
90−100
ZAR1

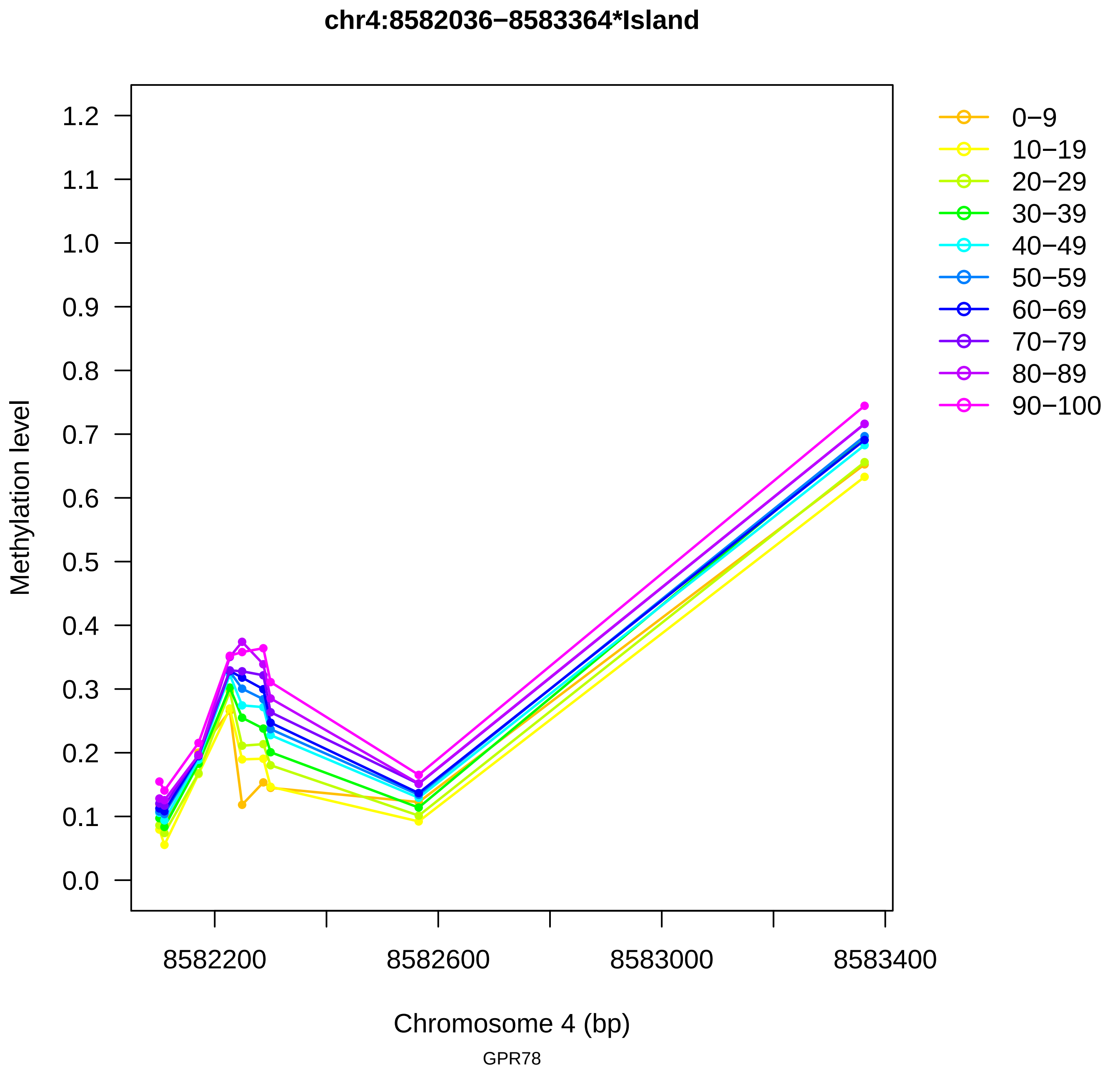
chr4:8582036−8583364*Island
Methylation level
0.0
0.1
0.2
0.3
0.4
0.5
0.6
0.7
0.8
0.9
1.0
1.1
1.2
8582200
8582600
8583000
8583400
Chromosome 4 (bp)
GPR78
0−9
10−19
20−29
30−39
40−49
50−59
60−69
70−79
80−89
90−100

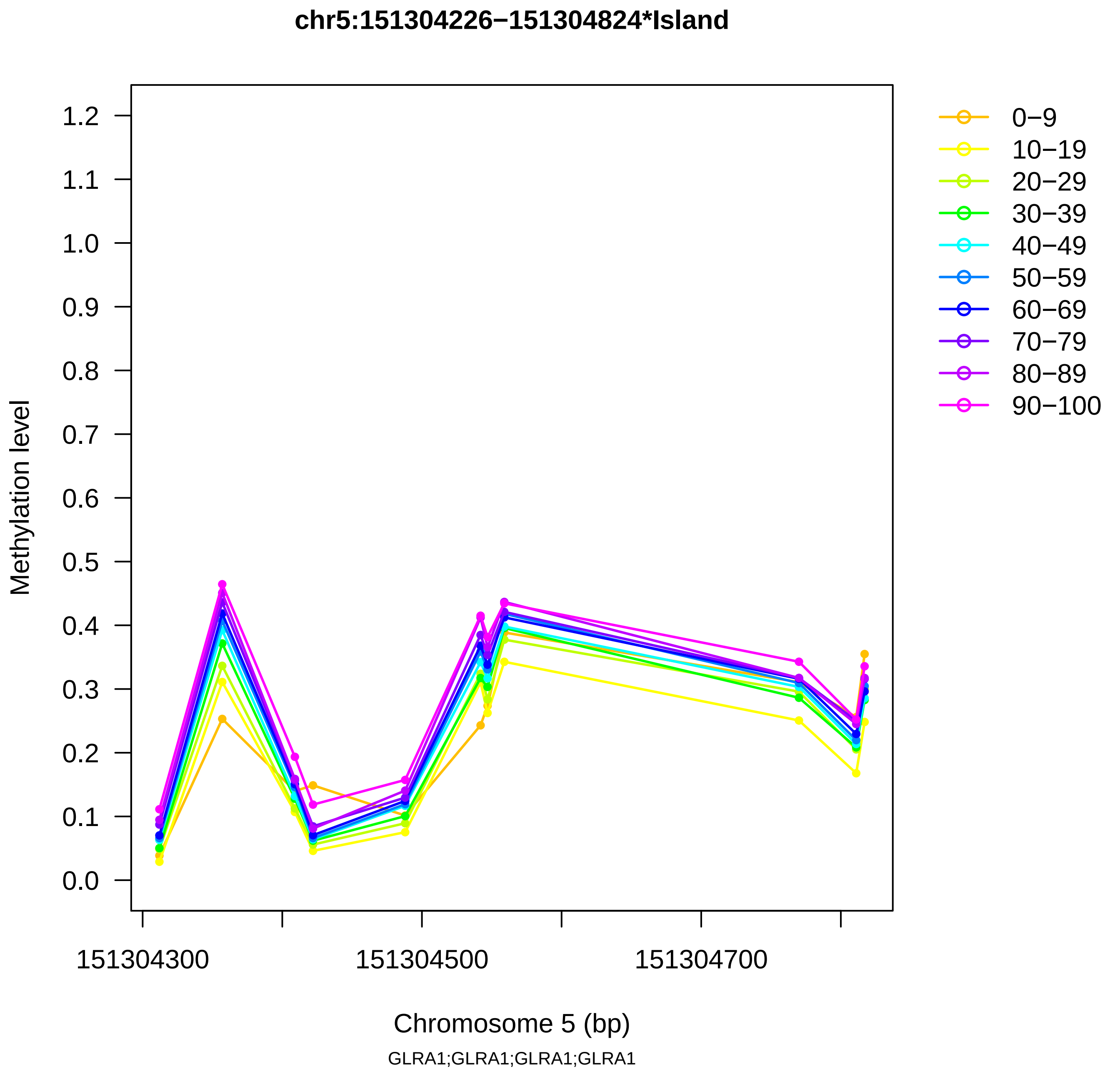

chr5:151304226−151304824*Island
Methylation level
0.0
0.1
0.2
0.3
0.4
0.5
0.6
0.7
0.8
0.9
1.0
1.1
1.2
151304300
151304500
151304700
Chromosome 5 (bp)
GLRA1;GLRA1;GLRA1;GLRA1
0−9
10−19
20−29
30−39
40−49
50−59
60−69
70−79
80−89
90−100

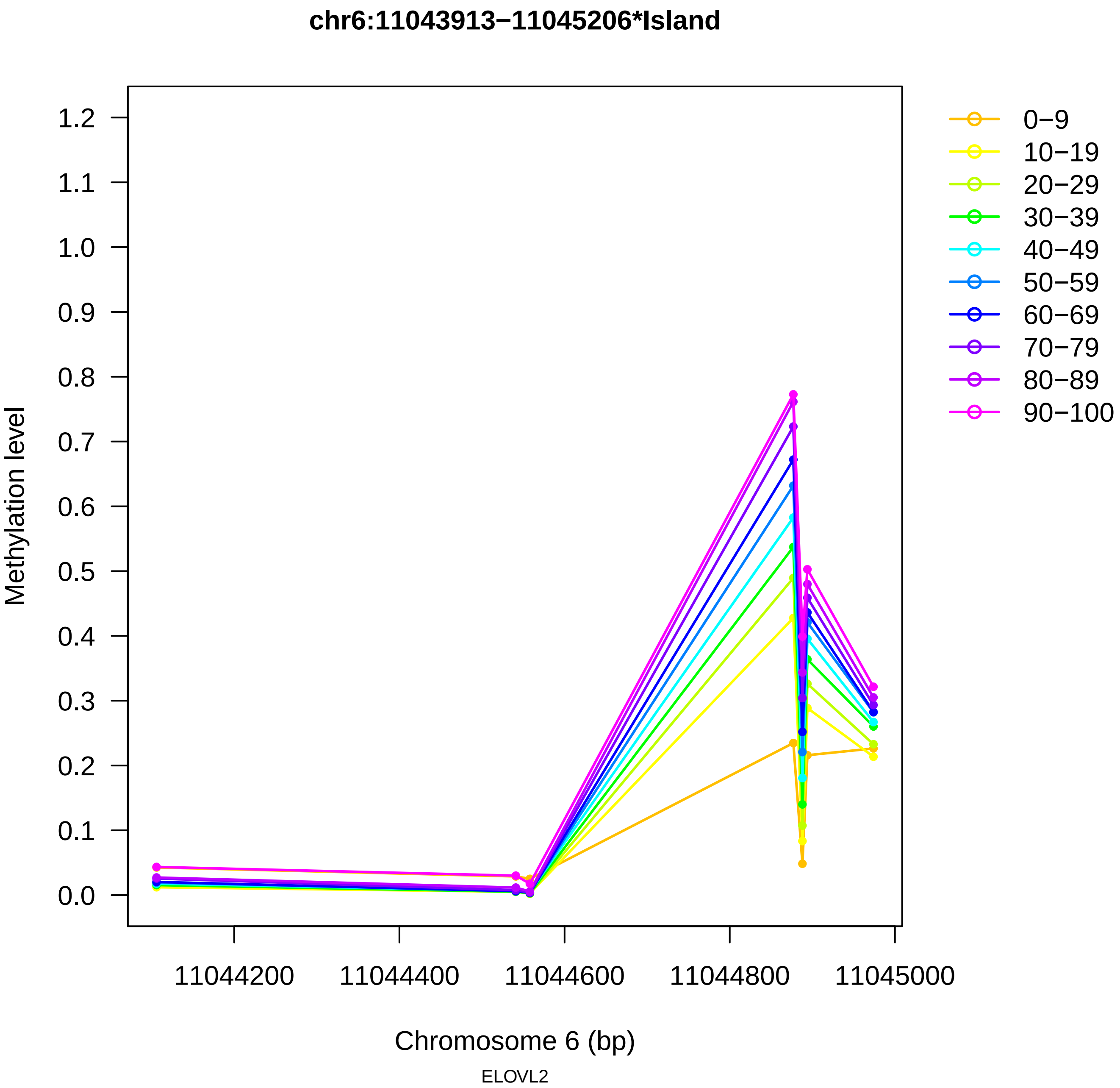

chr6:11043913−11045206*Island
Methylation level
0.0
0.1
0.2
0.3
0.4
0.5
0.6
0.7
0.8
0.9
1.0
1.1
1.2
11044200
11044400
11044600
11044800
11045000
Chromosome 6 (bp)
ELOVL2
0−9
10−19
20−29
30−39
40−49
50−59
60−69
70−79
80−89
90−100

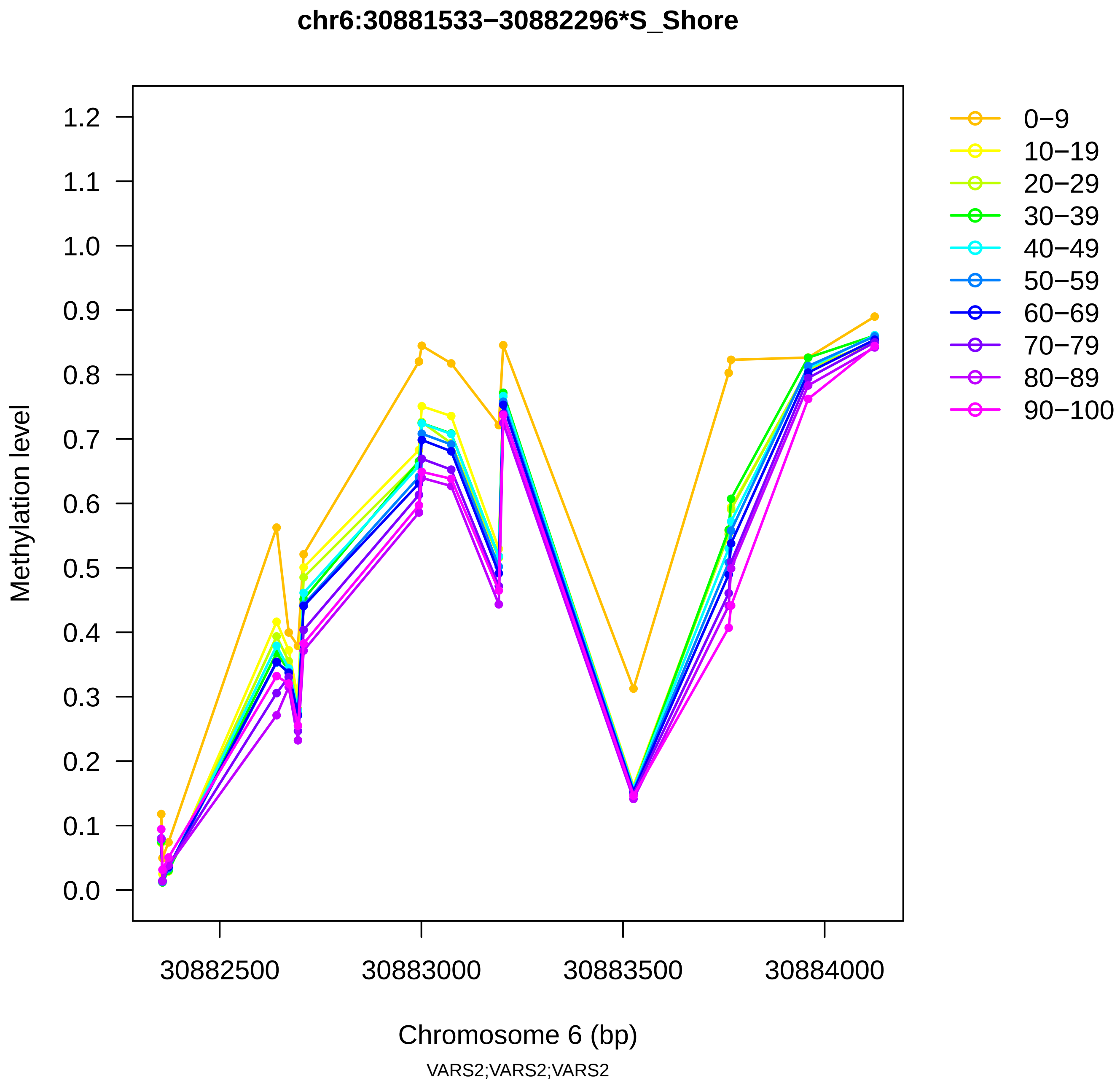
chr6:30881533−30882296*S_Shore
Methylation level
0.0
0.1
0.2
0.3
0.4
0.5
0.6
0.7
0.8
0.9
1.0
1.1
1.2
30882500
30883000
30883500
30884000
Chromosome 6 (bp)
VARS2;VARS2;VARS2
0−9
10−19
20−29
30−39
40−49
50−59
60−69
70−79
80−89
90−100

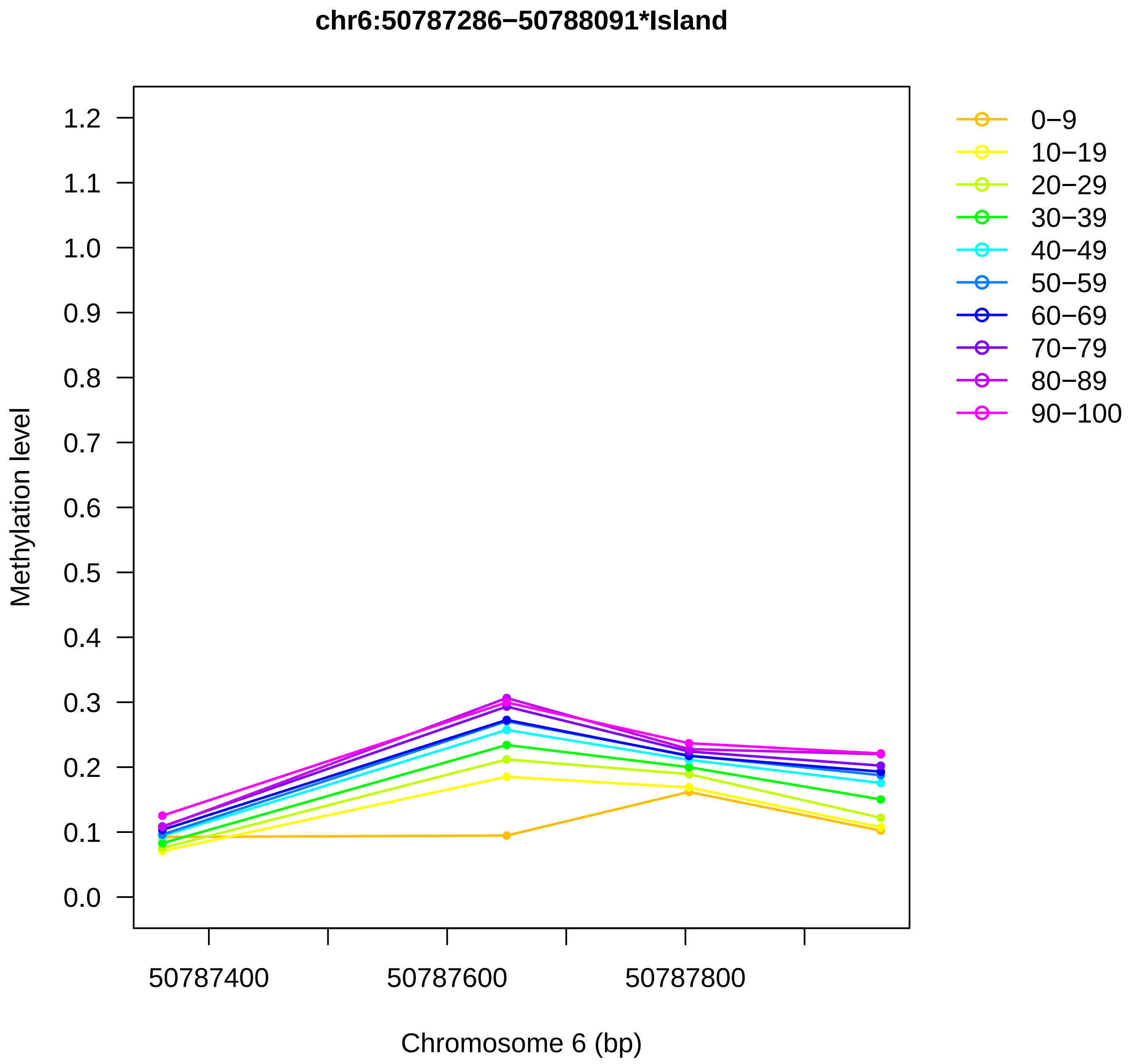
chr6:50787286−50788091*Island
Methylation level
1.2
1.1
1.0
0.9
0.8
0.7
0.6
0.5
0.4
0.3
0.2
0.1
0.0
50787400
50787600
50787800
Chromosome 6 (bp)
0−9
10−19
20−29
30−39
40−49
50−59
60−69
70−79
80−89
90−100

TFAP2B

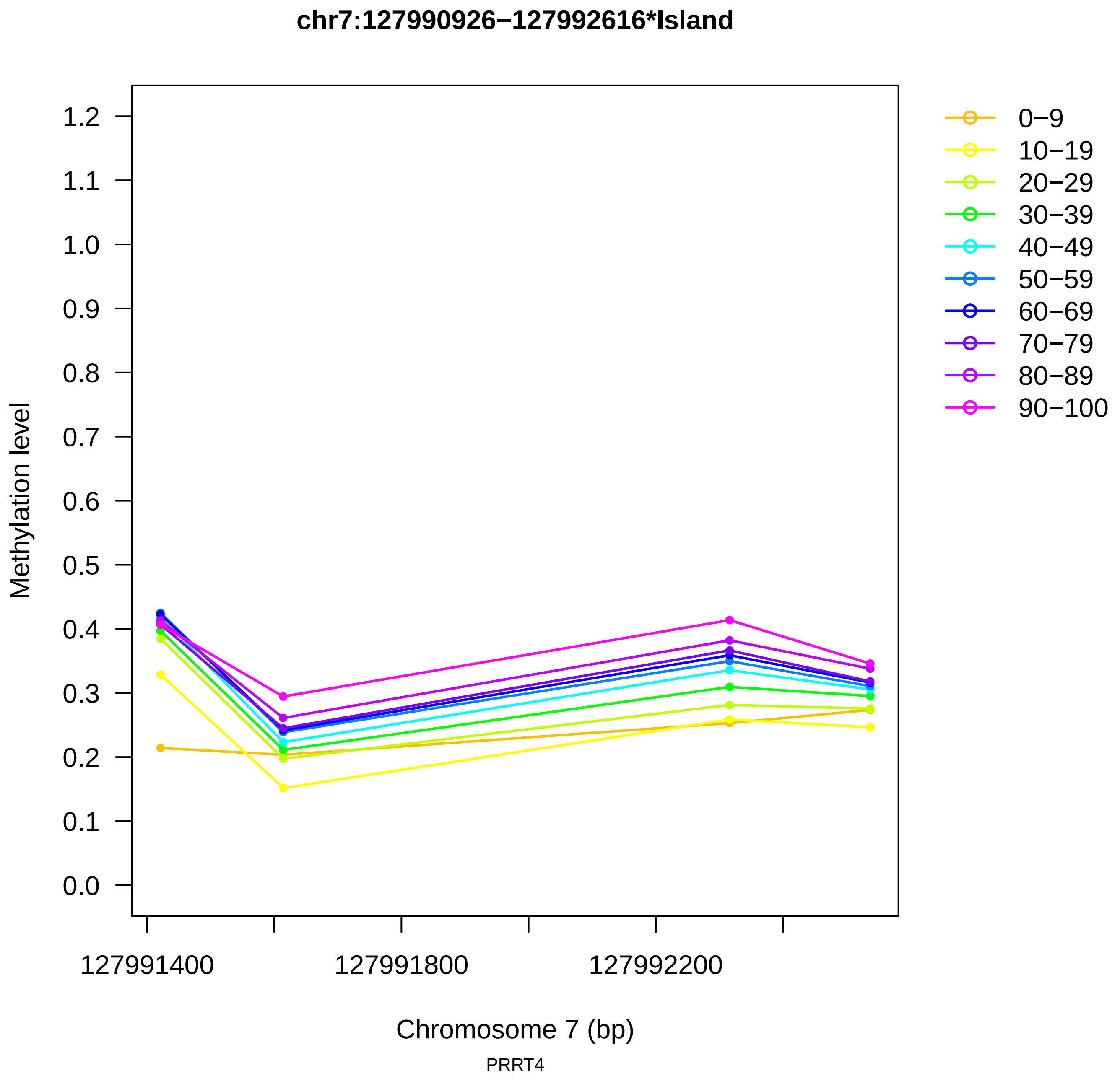
chr7:127990926−127992616*Island
Methylation level
1.2
1.1
1.0
0.9
0.8
0.7
0.6
0.5
0.4
0.3
0.2
0.1
0.0
127991400
127991800
127992200
Chromosome 7 (bp)
PRRT4
0−9
10−19
20−29
30−39
40−49
50−59
60−69
70−79
80−89
90−100

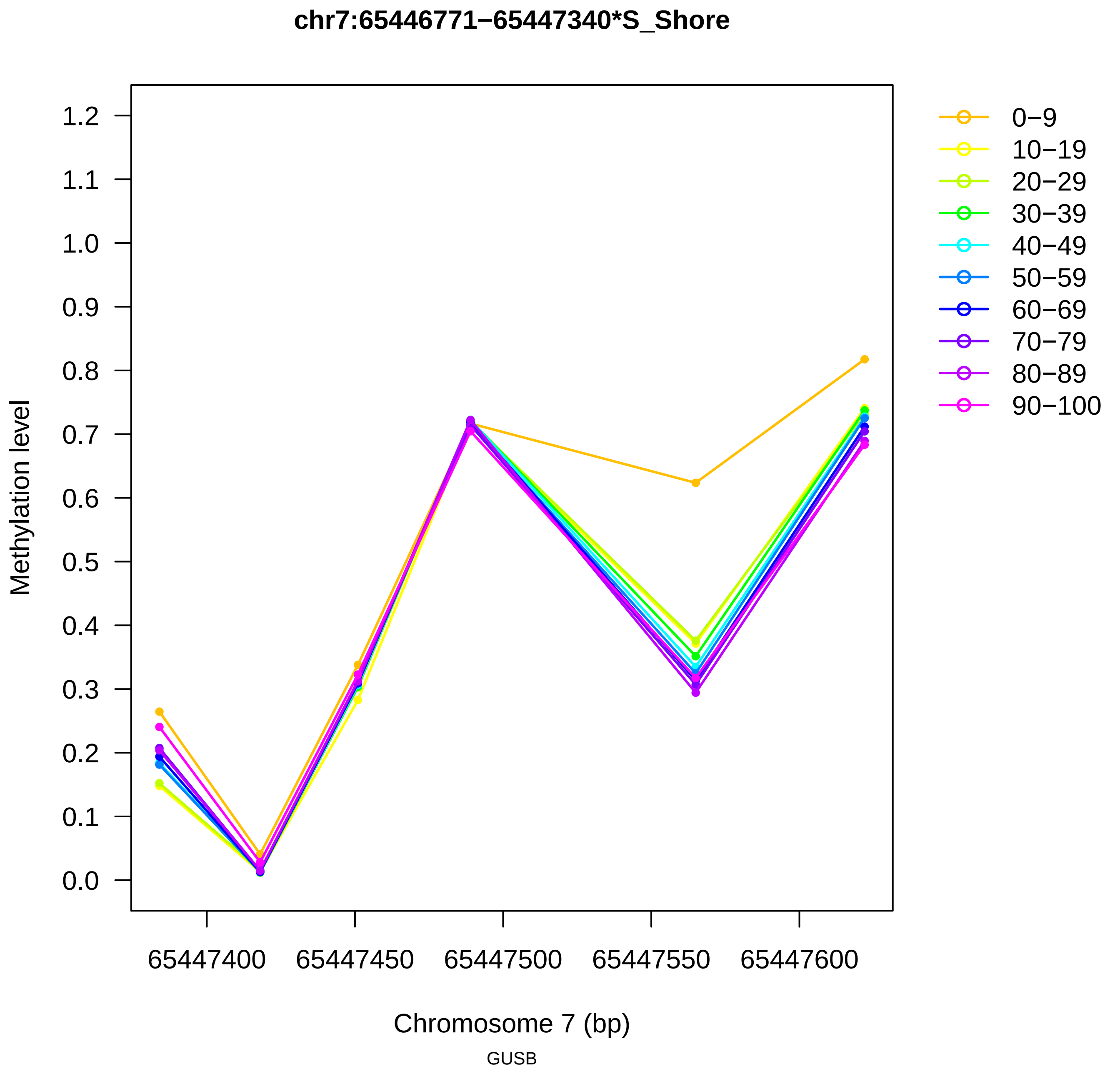
chr7:65446771−65447340*S_Shore
Methylation level
1.2
1.1
1.0
0.9
0.8
0.7
0.6
0.5
0.4
0.3
0.2
0.1
0.0
65447400
65447450
65447500
65447550
65447600
Chromosome 7 (bp)
GUSB
0−9
10−19
20−29
30−39
40−49
50−59
60−69
70−79
80−89
90−100

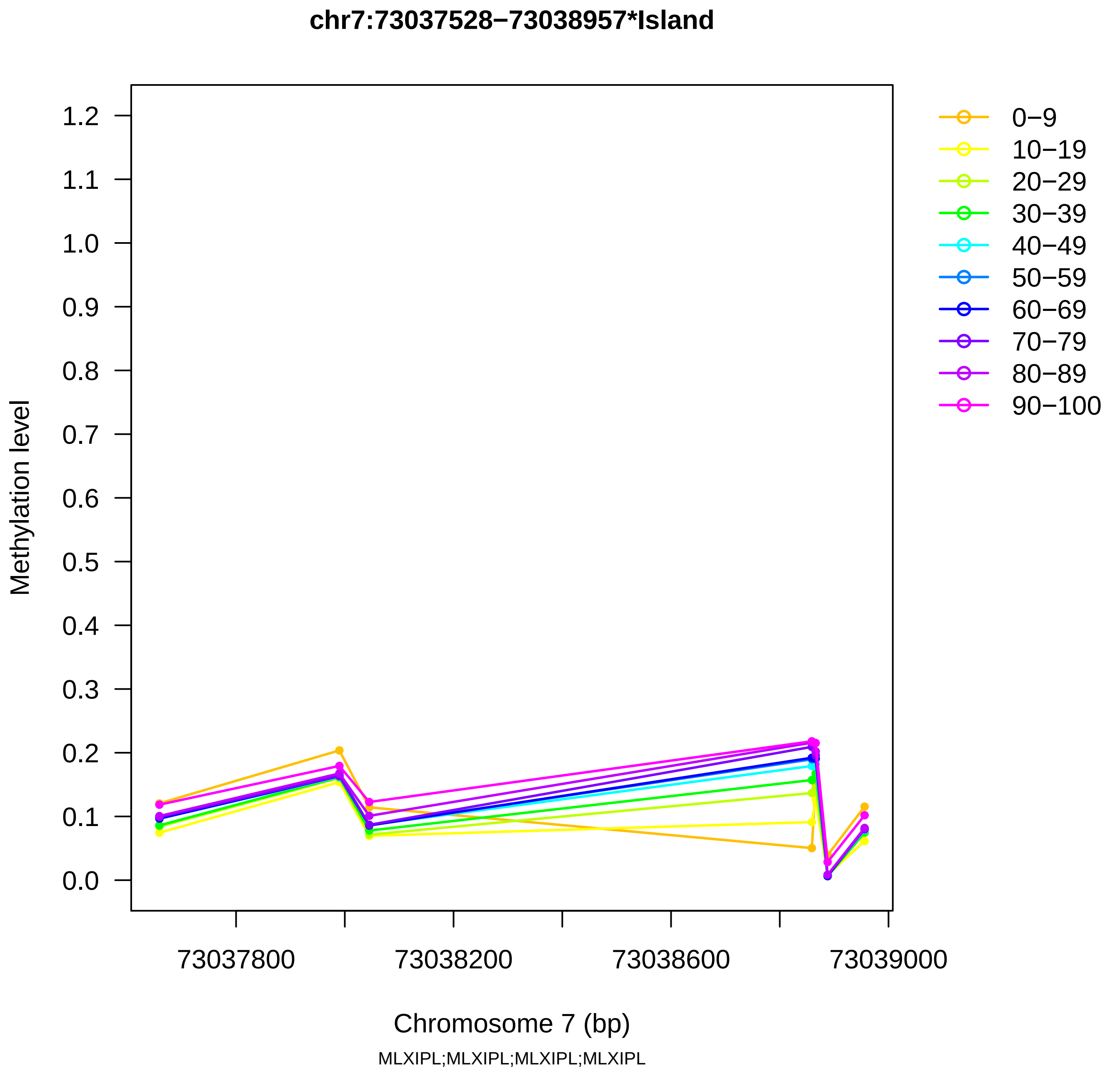

chr7:73037528−73038957*Island
Methylation level
1.2
1.1
1.0
0.9
0.8
0.7
0.6
0.5
0.4
0.3
0.2
0.1
0.0
73037800
73038200
73038600
73039000
Chromosome 7 (bp)
MLXIPL;MLXIPL;MLXIPL;MLXIPL
0−9
10−19
20−29
30−39
40−49
50−59
60−69
70−79
80−89
90−100

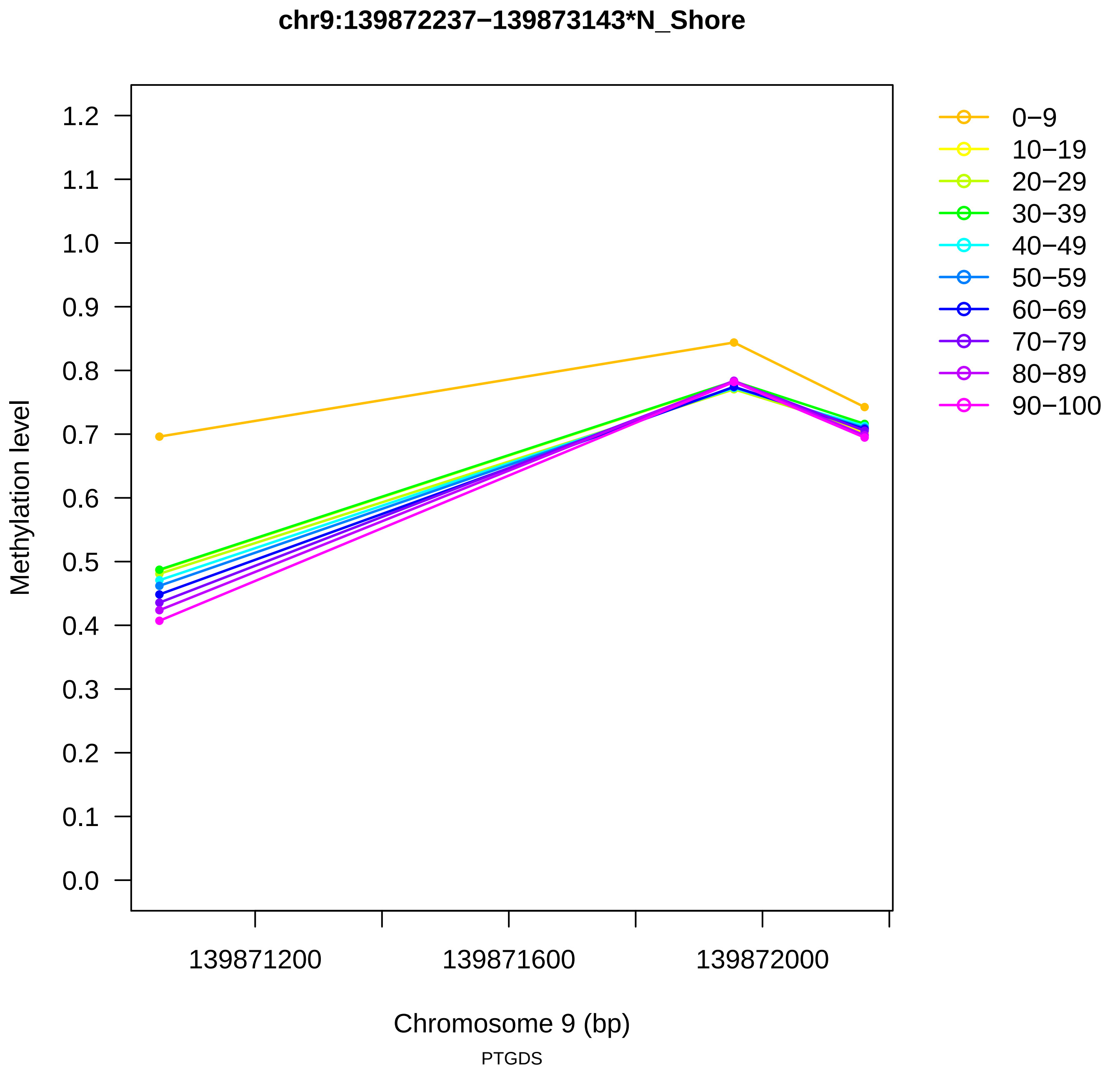
chr9:139872237−139873143*N_Shore
Methylation level
1.2
1.1
1.0
0.9
0.8
0.7
0.6
0.5
0.4
0.3
0.2
0.1
0.0
139871200
139871600
139872000
Chromosome 9 (bp)
PTGDS
0−9
10−19
20−29
30−39
40−49
50−59
60−69
70−79
80−89
90−100
